# NLRP3, NLRP6, and NLRP12 are inflammasomes with distinct expression patterns

**DOI:** 10.3389/fimmu.2024.1418290

**Published:** 2024-07-15

**Authors:** Bo Wei, Zachary P. Billman, Kengo Nozaki, Helen S. Goodridge, Edward A. Miao

**Affiliations:** ^1^ Department of Integrative Immunobiology, Duke University School of Medicine, Durham, NC, United States; ^2^ Department of Molecular Genetics and Microbiology, Duke University School of Medicine, Durham, NC, United States; ^3^ Department of Microbiology and Immunology, University of North Carolina at Chapel Hill, Chapel Hill, NC, United States; ^4^ Research Division of Immunology in the Department of Biomedical Sciences, Cedars-Sinai Medical Center, Los Angeles, CA, United States; ^5^ Board of Governors Regenerative Medicine Institute, Cedars-Sinai Medical Center, Los Angeles, CA, United States; ^6^ Department of Cell Biology, Duke University School of Medicine, Durham, NC, United States; ^7^ Department of Pathology, Duke University School of Medicine, Durham, NC, United States

**Keywords:** inflammasome, NLRP3, NLRP6, NLRP12, NLRP12 autoinflammatory disease

## Abstract

Inflammasomes are sensors that detect cytosolic microbial molecules or cellular damage, and in response they initiate a form of lytic regulated cell death called pyroptosis. Inflammasomes signal via homotypic protein-protein interactions where CARD or PYD domains are crucial for recruiting downstream partners. Here, we screened these domains from NLR family proteins, and found that the PYD domain of NLRP6 and NLRP12 could activate caspase-1 to induce cleavage of IL-1β and GSDMD. Inflammasome reconstitution verified that full length NLRP6 and NLRP12 formed inflammasomes *in vitro*, and NLRP6 was more prone to auto-activation. NLRP6 was highly expressed in intestinal epithelial cells (IEC), but not in immune cells. Molecular phylogeny analysis found that NLRP12 was closely related to NLRP3, but the activation mechanisms are different. NLRP3 was highly expressed in monocytes and macrophages, and was modestly but appreciably expressed in neutrophils. In contrast, NLRP12 was specifically expressed in neutrophils and eosinophils, but was not detectable in macrophages. NLRP12 mutations cause a periodic fever syndrome called NLRP12 autoinflammatory disease. We found that several of these patient mutations caused spontaneous activation of caspase-1 *in vitro*, which likely causes their autoinflammatory disease. Different cell types have unique cellular physiology and structures which could be perturbed by a pathogen, necessitating expression of distinct inflammasome sensors to monitor for signs of infection.

## Introduction

Pyroptosis is a form of regulated cell death that is proinflammatory and uniquely gasdermin-dependent (1). Pyroptosis exhibits large membrane balloons and occurs concomitantly with release of the proinflammatory cytokines IL-1β and IL-18. Inflammasomes are the sensors that most commonly initiate pyroptosis (2). Pathogen-associated molecular patterns (PAMPs) and host cell generated danger-associated molecular patterns (DAMPs) are recognized by germline encoded pattern recognition receptors (PRRs) (3). PRRs in the inflammasome family, such as NLRC4 and NLRP3, oligomerize and activate the downstream protease caspase-1 directly or through the adapter protein ASC (2, 4, 5). Multiprotein oligomerized platforms that activate caspase-1 are called inflammasomes. Once caspase-1 is activated, it cleaves the inflammatory cytokines pro-IL-1β and pro-IL-18 to their mature forms and additionally cleaves gasdermin D (GSDMD) (1, 2). After cleavage, the N-terminus of GSDMD moves to the plasma membrane, oligomerizes, and forms pores in the membrane with an inner diameter of 10 to 14 nm (1, 6, 7). These pores eventually cause pyroptosis in a process that also activates NINJ1 to form large membrane ruptures (8, 9). In parallel, caspase-4/5/11 act as sensors for cytosolic LPS and also cause pyroptosis (10–12). Pyroptosis is quite effective in preventing infection by environmental pathogens, and many host-adapted pathogens use virulence factors to inhibit pyroptosis in order to facilitate intracellular replication.

Bacterial infection can be sensed when type 3 secretion systems (T3SS) translocate flagellin, rod, or needle protein into the host cell cytosol. These three agonists directly bind to NAIP sensors that induce the oligomerization of NLRC4. This NLRC4 oligomer can directly interact with and activate caspase-1; additionally, this signaling can also be amplified through the adaptor ASC (13). In contrast, the NLRP3 inflammasome assembles in response to various stimuli including ATP, nigericin, and crystals (14). These stimuli cause cellular perturbations that, through mechanisms still being described, cause NLRP3 to oligomerize. This oligomerization requires help from NEK7, which acts as a structural co-factor that binds to NLRP3 (15–17). Unlike the NLRC4 inflammasome, NLRP3 oligomers cannot activate caspase-1 directly, but must signal through the adapter protein ASC.

Both NLRC4 and NLRP3 belong to the NLR superfamily. Based on differences in their N-terminus, NLR proteins are divided into NLRA, NLRB, NLRC, NLRP, and NLRX subfamilies (18) (**Supplementary Figure 1A**). Of these, several NLRs form inflammasomes, including NLRB, NLRC, and NLRP family members, which contain N-terminal BIR domains, caspase recruitment domains (CARDs), or pyrin domains (PYDs), respectively. NAIPs are in the NLRB family, however the BIR domains do not bind to downstream signaling proteins, instead the co-polymerizing NLRC4 contains the CARD domain of the NAIP/NLRC4 inflammasome (13) (**Supplementary Figure 1B**). NLRP3 contains a PYD domain that confers downstream signaling to this inflammasome (2) (**Supplementary Figure 1C**).

Interactions between NLRs, the adaptor protein ASC, and caspase-1 are mediated by homotypic protein-protein interactions of their CARD or PYD domains, which are members of the death domain superfamily (19). Thus, a specific CARD will interact with another cognate CARD (CARD-CARD interactions); similarly, homotypic PYD-PYD interactions also occur (19). However, not every CARD interacts with every other CARD, rather, the interactions are specific. For example, the CARD of NLRC4 interacts with the CARD of ASC or the CARD of caspase-1 (13), but not with the CARD of the type I interferon signaling protein MAVS. As an adaptor protein, ASC contains both a PYD and a CARD that bridges upstream PYD-containing sensors to the CARD of caspase-1. Additionally, ASC can also be recruited to CARD-containing sensors to amplify their signaling. While the protein-protein interactions of the NLRC4 and NLRP3 inflammasomes have been well-characterized, the interactions for other NLR family members remain poorly defined. Here, we search for additional inflammasomes by screening NLR family members for their ability to signal to ASC and/or caspase-1.

## Material and methods

### Antibodies and reagents

The following antibodies were used: mouse monoclonal anti-FLAG M2 antibody (F1804, SIGMA); mouse monoclonal anti-HA (16B12) (MMS-101P, Covance); myc antibody(9E10) (sc-40, Santa Cruz); Peroxidase-conjugated AffiniPure Goat Anti-Mouse IgG (H+L) (115–035-062, Jackson ImmunoResearch); Goat anti-Mouse IgG (H+L) Highly Cross-Adsorbed Secondary Antibody, Alexa Fluor™ Plus 555 (A32727, Invitrogen).

The following reagents were used: AP20187 (SML2838, SIGMA); Nigericin (tlrl-nig-5, InvivoGen); TRIzol reagent (15596026, Invitrogen); SuperScript™ II Reverse Transcriptase (18064014, Invitrogen); RNaseOUT™ Recombinant Ribonuclease Inhibitor (10777019, Invitrogen); Deoxynucleotide (dNTP) Solution Mix (N0447L, NEB); Dimethyl sulfoxide (DMSO) (D4540, SIGMA); Retinoic acid (ATRA) (R2625, SIGMA); Phorbol 12-myristate 13-acetate (PMA) (tlrl-pma, InvivoGen); (Z)-4-Hydroxytamoxifen (4-OHT) (74052, STEMCELL); Recombinant Murine GM-CSF (315–03, Peprotech); Phenylmethanesulfonyl fluoride solution (PMSF) (93482, SIGMA); Protease Inhibitor Cocktail (HY-K0010, MCE); Puromycin (P8833, SIGMA); RNAlater Stabilization Solution(AM7020, Invitrogen); PowerTrack SYBR Green Master Mix (A46110, Invitrogen); Polybrene (H9268, SIGMA); Paraformaldehyde (PFA) (P6148, SIGMA); DAPI (D8417, SIGMA).

### Cell culture

HEK293T/17 (CRL-11268, ATCC), HeLa (CCL-2, ATCC), COS-1 (CRL-1650, ATCC) and L-929 (CCL-1, ATCC) cells were maintained in DMEM (11995073, Gibco) supplemented with 1% Penicillin-Streptomycin (15140122, Gibco) and 10% Fetal Bovine Serum (SH30396.03, Cytiva). HL-60 (CCL-240, ATCC) cell was maintained in RPMI1640 (11875093, Gibco) supplemented with 1% Penicillin-Streptomycin (15140122, Gibco) and 10% Fetal Bovine Serum (10082147, GIBCO). For HL-60 cell neutrophil differentiation, cells were induced with a final concentration of 1.25% DMSO plus 1 μM ATRA for 6 days. For HL-60 cell macrophage differentiation, cells were induced with 20 nM PMA for at least 3 days. Hoxb8 progenitor cells were maintained in RPMI1640 (11875093, Gibco) supplemented with 1% Penicillin-Streptomycin (15140122, Gibco), 20 ng/mL, GM-CSF, 100 nM 4-OHT and 10% Fetal Bovine Serum (10082147, GIBCO). For Hoxb8 progenitor cell differentiation, cells were induced by withdrawing 4-OHT or L-cell medium, which contained DMEM, 10% L-929 cell culture medium and 10% Fetal Bovine Serum (SH30396.03, Cytiva). All the cell lines were tested regularly for mycoplasma infection by PCR.

### qRT-PCR

Tissue samples from C57/B6J mice (about 20μg) or cultured cells (about 4×10^6^ cells) were harvested for RNA extraction with TRIzol reagent. For tissue samples, homogenization was essential. 1ug total RNA was subjected to reverse transcription through SuperScript II Reverse Transcriptase. The gene expression was assayed by normal SYBR green method with PowerTrack SYBR Green Master Mix. The results were analyzed by ΔΔCT method. The mouse tissue samples could be stored in RNAlater Stabilization Solution at -80°C if the experiments were not processed immediately. The cDNA samples could be stored at -80°C, too. The PCR primers were designed by Primer-Blast (https://www.ncbi.nlm.nih.gov/tools/primer-blast/), sequence as follow: mouse 18S rRNA (forward: GGCCGTTCTTAGTTGGTGGA, reverse: TCAATCTCGGGTGGCTGAAC); mouse *Gapdh* (forward: GAAGGTCGGTGTGAACGGAT, reverse: TTCCCATTCTCGGCCTTGAC); mouse *Nlrp6* (forward: AGCTGTAGAAATGACCCGGC, reverse: GAACGCTGACACGGAGAGAA); mouse *Nlrp3* (forward: AGAGTGGATGGGTTTGCTGG, reverse: CGTGTAGCGACTGTTGAGGT); mouse *Nlrp12* (forward: TGGCTCTCAGCACCTTTCAG, reverse: AGAGACATCCAAAGGGCACG); human *GAPDH* (forward: GGAAGGTGAAGGTCGGAGTC, reverse: TGGAATTTGCCATGGGTGGA): human *NLRP6* (forward: ACCACAAAACAACTGCCAGC, reverse: CCTCAGGGCCTCAGAAAGGT); human *NLRP3* (forward: CACTGTCCCTGGGGTTTCTC, reverse: CCCGGCAAAAACTGGAAGTG); human *NLRP12* (forward: TGTGGGAGAGAGGACAGAGAG, reverse: AGGTTTCCTGGGGATCTTTTCT).

### Transfection and immunoblotting analysis

The HEK293T/17 cells were transiently transfected with Calcium Phosphate Transfection method (20). The reagents were 2.5 M CaCl_2_ and 2× HEPES buffer (50 mM HEPES, pH 7.05, 280 mM NaCl, 1.5 mM Na_2_PO_4_). Medium was changed with fresh medium 8 hours post transfection. The cells were harvested with DPBS (14190144, Gibco), and lysed with RIPA buffer (50 mM Tris, pH 7.4, 150 mM NaCl, 1% Triton, 0.1% sodium deoxycholate, 0.01% SDS, 1 mM EDTA, 1 mM EGTA, 2 mM NaF, 1 mM Na3VO4, 1 mM β-glycerophosphate, 1 mM PMSF, protease inhibitor cocktail). The cell lysates were quantified by BCA methods, and about 15ug total protein was loaded into each lane of an SDS PAGE gel. These were subjected to western blotting with the indicated antibodies.

### Plasmids

Mouse NLRP3 and NEK7 expression plasmid were purchased from addgene (Addgene #75127 and #75142). Mouse NLRP12 plasmid was a gift from Dr. Hasan Zaki. Mouse IL-1β was cloned into pEF6 (Thermo) with C-terimal flag tag, while mouse GSDMD was cloned into pLenti-EF1a-IRES-Puro (a gift from Dr. Youssef Aachoui) with N terminal Myc tag. Mouse Caspase-1 was inserted into pMXs-IRES-Puro (Cell Biolabs), while mouse ASC was inserted into pEF6-V5(Thermo). Mouse NLRP6 was cloned into pLenti-EF1a-IRES-Puro with N-terminal flag tag. The CARD domain or the N terminal of mouse *Apaf* (1–97Aa), *Aire* (1–100Aa), *Ciita* (1–90Aa), *Nlrc3* (1–175Aa), *Nlrc4* (1–89Aa), *Nlrc5* (1–92Aa), *Nlrx1*(1–101Aa), the PYD domain from mouse *Nlrp2* (1–95Aa), *Nlrp3* (1–91Aa), *Nlrp4a* (1–89Aa)*, Nlrp4b* (1–93Aa)*, Nlrp4c* (1–89Aa)*, Nlrp4d* (1–93Aa)*, Nlrp4e* (1–89Aa), *Nlrp4f* (1–92Aa), *Nlrp4g* (1–93Aa), *Nlrp6* (1–91Aa), *Nlrp9a* (1–93Aa), *Nlrp9b* (1–91Aa)*, Nlrp9c* (1–91Aa), *Nlrp10* (1–92Aa), *Nlrp12* (1–96Aa), *Nlrp14* (1–80Aa), and the PYD domain from human *NLRP1*(1–93Aa), *NLRP2* (1–94Aa), *NLRP3* (1–94Aa), *NLRP4* (1–94Aa), *NLRP5* (1–97Aa), *NLRP6* (11–103Aa), *NLRP7* (1–93Aa), *NLRP8* (1–123Aa), *NLRP9* (1–94Aa), *NLRP10* (1–96Aa), *NLRP11* (1–91Aa), *NLRP12* (1–95Aa), *NLRP13* (1–107Aa), *NLRP14* (1–96Aa), were subcloned into pC4M-Fv2E (Clontech).

### Stable cell line construction

The cDNA of *Nlrp3* and *Nlrp12* were subcloned into lentiviral expression vector pLenti -EF1a-C-Myc-DDK-IRES-Puro (a gift from Dr. Youssef Aachoui). Lentiviral package process was followed the addgene protocol (https://www.addgene.org/protocols/lentivirus-production/). Briefly, the expression plasmid, together with viral package plasmid psPAX2 and pMD2.G, were transfected into HEK293T/17 cells at the ration of 4:3:1. Medium that contained lentiviral particles, was harvested 2 days later, and filtered by 0.45μM low protein binding filter. Hela, COS-1 and HL-60 cells were transduced with lentiviral particles in the presence of polybrene (5μg/mL). The transduced cells were selected by puromycin(2μg/mL) 2 days later for 1 week. The protein expression was detected by immunoblotting.

### Mouse sample collection

Mouse strains were bred and housed at Duke University in specific pathogen free facility. Animal protocols were approved by the Institutional Animal Care and Use Committee (IACUC) at Duke University and met guidelines of the US National Institutes of Health for the humane care of animals. The wild type C57B/J mice were euthanized, and blood, bone marrow, kidney, liver, lung, spleen, thymus, small intestine, large intestine were collected. Intestinal epithelial cells (IECs) were isolated from 10-to-15-week-old wild-type mice as previously described (21, 22). After euthanasia, the intestine was removed and cut open, then washed three times. Peyer’s patches were eliminated to avoid immune cell contamination. This is followed by 2.5 mM EDTA chelation for 30 min at 4 °C and mechanical dissociation. The isolated IECs were pelleted and washed three times in 2% sorbitol PBS with low-speed centrifugation (400 rpm, 3 min) at 4 °C to further exclude immune cells, and were resuspended in 1% FCS/DMEM. After being filtered through 70-μm strainers, RNA isolation was performed.

### AlphaFold prediction

The structure of mouse NLRP3 (UniProt: Q8R4B8) and NLRP12 (UniProt: E9Q5R7) were from AlphaFold database; the peptide structures were predicted via colabFolad notebook (23, 24).

### Phylogenetic analysis of mouse NLRPs proteins

NLRP protein sequences were downloaded from NCBI. The tree was constructed using BEAST 1.10.4. Sequences were aligned using CLUSTALO 1.2.4. MCMC chain length was set to 10 million steps. Phylogenies were derived using 4 parameter gamma heterogeneity using the LG model for amino acid substitution. The consensus tree was derived from resultant trees using the most likelihood estimate.

### RNAseq analysis

Analysis of *Nlrp3* and *Nlrp12* expression by hematopoietic progenitors and monocytes was performed using a published RNAseq dataset (GEO: GSE88982) of cells isolated from mouse bone marrow (25)(https://pubmed.ncbi.nlm.nih.gov/29166589/). Ex vivo GMP, GP and MDP, as well as monocyte-committed progenitors and classical monocytes derived from GMP and MDP *in vitro*, were analyzed by RNAseq.

### Immunofluorescence

About 8x 10^4^ cells were plated on circular cover glasses (12–541-001, Fisherbrand) in 24 well plates. After 24 hours, the cells were treated with Nigericin (20 μM) for 60 min or not. Then, cells were processed as follow: fixed by 4% PFA (pH 7.4 in DPBS) for 15 min; permeabilized by 0.25% Triton X-100 in PBS for 15 min; blocked by blocking buffer (5% normal goat serum in PBS-0.05% Tween 20 (PBS-T)) for 1hour; immunostained overnight with indicated primary antibodies in a humidified chamber at 4°C; washed 3 times with PBS-T; subsequently incubated with Alexa Fluor Plus 555 -conjugated secondary antibodies for 1 h; washed 3 times again and stained with DAPI (10 μg/mL) for 10 min; Mounted on the slide with anti-fade mounting medium. Images were captured on Zeiss LSM 780, and analysis by ImageJ (Fiji).

## Results

### Identify CARD and PYD domains that activate caspase-1

**Figure 1:**
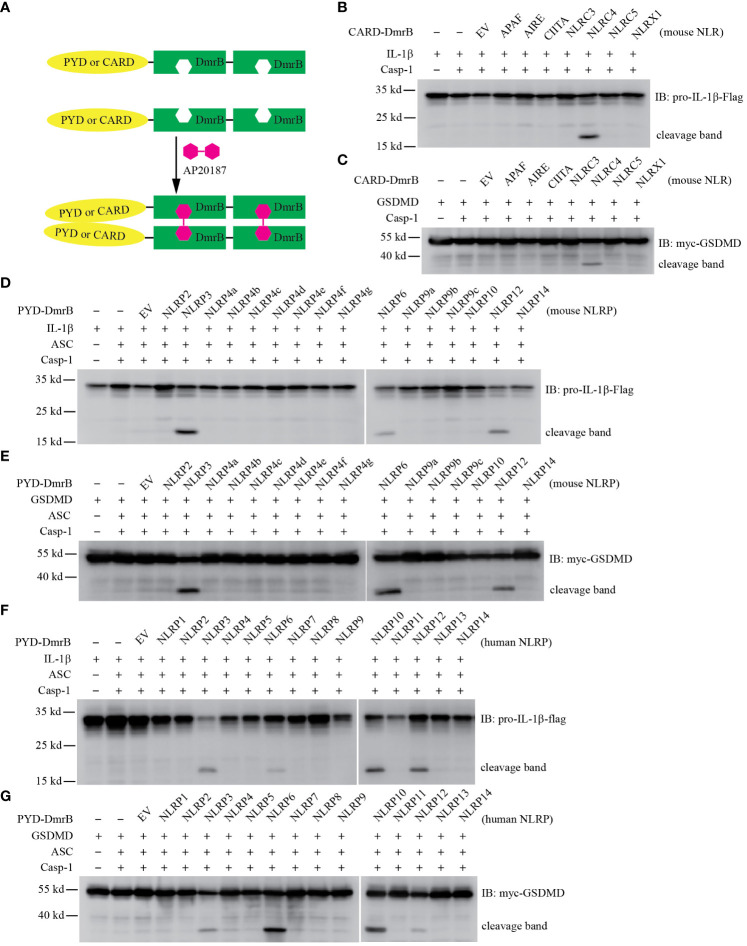
Screening CARD and PYD domains by inducible dimerization. **(A)** Diagram of AP20187 induced dimerization experiment system. **(B-G)** HEK293T/17 cells were transiently transfected with indicated plasmids. 24 hours later the dimer inducer AP20187 (20 nM) was added for another 10 hours. Cells were harvested and lysed, then lysates were subjected to immunoblotting with indicated antibody. EV, empty vector. **(B, C)** Mouse CARD domain fusion proteins did not induce cleavage of IL-1β **(B)** and GSDMD **(C)** through Caspase-1. The CARD domain from *Apaf*, *Aire*, *Ciita*, *Nlrc3*, and *Nlrc4*, as well as the N-terminal domain of *Nlrx1* were expressed as fusion proteins with tandem DmrB domains. **(D, E)** Mouse NLRP6 and NLRP12 PYD domain fusion proteins induced cleavage of IL-1β **(D)** and GSDMD **(E)**. The PYD domain from *Nlrp2*, *Nlrp3*, *Nlrp4a, Nlrp4b, Nlrp4c, Nlrp4d, Nlrp4e*, *Nlrp4f*, *Nlrp4g*, *Nlrp6*, *Nlrp9a*, *Nlrp9b, Nlrp9c*, *Nlrp10*, *Nlrp12*, and *Nlrp14* were expressed as fusion proteins with tandem DmrB domains. **(F, G)** Human NLRP6, NLRP10 and NLRP12 PYD domain fusion proteins induced cleavage of IL-1β **(F)** and GSDMD **(G)**. The PYD domain from *NLRP1*, *NLRP2*, *NLRP3*, *NLRP4*, *NLRP5*, *NLRP6*, *NLRP7*, *NLRP8*, *NLRP9*, *NLRP10*, *NLRP11*, *NLRP12*, *NLRP13*, and *NLRP14* were expressed as fusion proteins with tandem DmrB domains. All the blotting results are representative of at least 3 independent experiments.

We wondered whether other proteins that also contain CARD or PYD could activate caspase-1 via homotypic protein-protein interactions. We chose to use the chemically inducible dimerization system, where protein-protein interactions are specifically induced by addition of dimerizing drug (**Figure 1A**, **Supplementary Figures 1C, D**). The CARD and PYD domains of interest were fused to the DmrB binding domain, which originates from the FK506-binding protein FKBP12. Homo-dimerization is induced by the cell-permeable ligand AP20187 (also called B/B homodimerizer). This method has been previously used to study inflammasome signaling (26), where DmrB dimerization simulates the oligomerization of an NLR inflammasome. We first optimized our transfection conditions to avoid autoactivation of ASC and caspase-1.

We first examined proteins with CARD domains to determine whether they activate caspase-1. We transfected HEK293T/17 cells with IL-1β, caspase-1, and the CARD-DmrB construct, and assessed IL-1β cleavage by western blotting. We used the CARD domain of NLRC4 as a positive control (13), and confirmed that fusion of the NLRC4 CARD to DmrB and dimerization with AP20187 activates caspase-1 and causes IL-1β cleavage (**Figure 1B**). In the absence of AP20187 we observed notably weaker IL-1β cleavage (data not shown). We next tested the CARD domains from other proteins that are not known to activate caspase-1: APAF, AIRE, CIITA, NLRC3, and NLRC5 (18) (**Supplementary Figure 1E**). In contrast to NLRC4, none of these CARD-DmrB fusion proteins induced cleavage of IL-1β (**Figure 1B**), though they were all expressed at relatively similar levels (**Supplementary Figure1E**). Although it is not classified as a CARD domain, we also tested the N-terminal domain of NLRX1, which also failed to induce IL-1β cleavage (**Figure 1B**). Similarly, the processing of GSDMD was only induced by the NLRC4 CARD fusion protein, but none of other proteins (**Figure 1C**).

Next, we examined PYD domains from mouse NLRP proteins (18) (**Supplementary Figure 1B**). In the classical NLRP3 inflammasome, NLRP3 recruits the adaptor protein ASC through PYD-PYD interactions (2). We used the NLRP3 PYD as a positive control and verified that dimerization accomplishes IL-1β cleavage (**Figure 1D**). We next tested the PYD domains of all other NLRP proteins from mice (**Supplementary Figure 1F**). Interestingly, the NLRP6 PYD and the NLRP12 PYD fusion proteins also induced IL-1β cleavage, whereas the other PYD fusion proteins which originated from NLRP2, NLRP4a, NLRP4b, NLRP4c, NLRP4d, NLRP4e, NLRP4f, NLRP4g, NLRP6, NLRP9a, NLRP9b, NLRP9c, NLRP10, or NLRP14 did not cause any cleavage of IL-1β (**Figure 1D**). Similarly, only the NLRP3, NLRP6, and NLRP12 PYD fusion proteins resulted in cleavage of GSDMD (**Figure 1E**). To our surprise, our results did not validate two recently described inflammasomes. NLRP9b has been reported to recognize short double-stranded RNA stretches via RNA helicase DHX9 and form inflammasome complexes together with the adaptor proteins ASC and caspase-1 (27). NLRP10 has been reported to monitor mitochondrial integrity in an mtDNA-independent manner, and form inflammasome with ASC to activate caspase-1 (28, 29). However, their PYD domains did not result in IL-1β cleavage or GSDMD cleavage in our inducible dimerization system. Therefore, this system may be susceptible to false-negative results.

In order to determine whether these results with murine proteins hold true in humans, we next studied the PYD domains from human NLRP proteins (18) (**Supplementary Figure 1G**). Similar to the results from the mouse homologs, the PYD from NLRP3, NLRP6, and NLRP12 resulted in cleavage of IL-1β and GSDMD, while neither IL-1β nor GSDMD were inducibly cleaved by NLRP1, NLRP2, NLRP4, NLRP5, NLRP7, NLRP8, NLRP9, NLRP11, NLRP13, or NLRP14 (**Figures 1F, G**). To our surprise again, the human NLRP7 PYD did not result in cleavage of IL-1β, although NLRP7 has been reported to recognize microbial lipopeptides in human macrophage and assemble inflammasome (30). Interestingly, we found human NLRP10 PYD fusions did result in cleavage of IL-1β and GSDMD, whereas the PYD domain of mouse NLRP10 could not (**Figures 1D, G**). This difference may be due to false-negative results with the murine NLRP10, or could be caused by the absence of murine co-factors that are essential but which do not exist in human HEK293T/17 cells. PYD and CARD domains are highly diverse with no specific identifying sequence or motif, and thus individual PYD or CARD domains could be subject to specific regulatory modification. For example, the NLRP3 PYD domain can be post translationally modified by phosphorylation, acetylation, or other modifications (31, 32). It may be that an absent posttranslational modification of PYD domain of NLRP7, NLRP9b, and mouse NLRP10 caused a false negative result in our assay.

Taken together, we observed that in addition to NLRP3, the PYD domains of NLRP6, NLRP10, and NLRP12 activate caspase-1 and result in cleavage of IL-1β and GSDMD. We chose to further study NLRP3, NLRP6, and NLRP12.

### Reconstitution of the NLRP3 inflammasome

**Figure 2:**
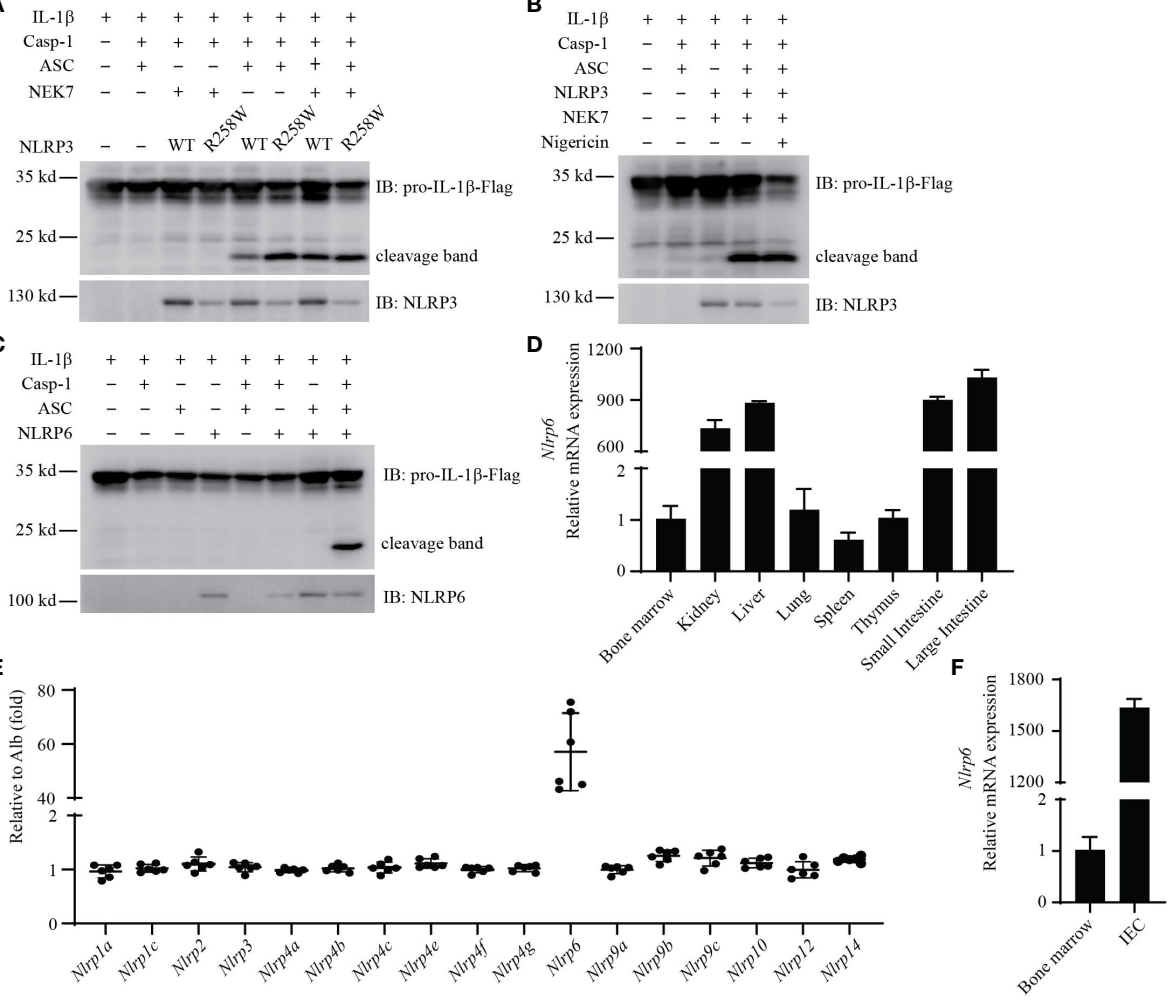
Reconstitution of mouse NLRP3 and NLRP6 inflammasome *in vitro.* ***(*A, B*)*** Reconstitution of the NLRP3 inflammasome in HEK293T/17 cells with IL-1β, caspase-1, ASC, NEK7, and either NLRP3 (WT) or NLRP3(R258W). **(B)** Addition of nigericin (20 μM) treatment for 90min. Cleaved IL-1β was detected by western blot. **(C)** Reconstitution of the NLRP6 inflammasome in HEK293T/17 cells with IL-1β, caspase-1, ASC, and NLRP6. Cleaved IL-1β was detected by western blot. **(D)** qRT-PCR analysis of *Nlrp6* expression in indicated mouse tissues. **(E)** The relative expression of *Nlrp* genes in IECs. Data extracted from the original data in NCBI (GDS3921). The expression results were normalized by albumin (*Alb*) expression as a gene that should not be expressed (thus a value of 1 reflects absent expression). **(F)** qRT-PCR analysis of *Nlrp6* expression in purified IECs compared to bone marrow cells. All the results are representative of at least 3 independent experiments.

NLRP3 inflammasome signaling can be reconstituted in HEK293T cells (33), and we wanted to use this approach to study NLRP6 and NLRP12. First, we optimized the NLRP3 reconstitution as a positive control. Because our laboratory has more experience studying murine inflammasomes, we chose to first study the murine genes. We used HEK293T cells as a generic highly transfectable cell type. We first choose to detect cytosolic IL-β cleavage in live cells by western blot. We optimized the system with NLRP3, and used optimized transfection conditions where ASC and caspase-1 alone did not cause autoactivation (**Figure 2A**). Expression of full length of NLRP3 together with ASC and caspase-1 resulted in cleavage of IL-1β by western blot (**Figure 2A**). The amount of NLRP3 we transfected resulted in autoactivation without application of specific NLRP3 agonists (**Figure 2A**).

We next validated that the NLRP3 hyperactivation mutant R258W resulted in enhanced cleavage of IL-1β compared to WT NLRP3 (34) (**Figure 2A**). Interestingly, ectopic expression of NEK7 increased the cleavage of IL-1β in cells expressing WT NLRP3 (**Figure 2A**, lane 5 and lane 7), indicating that endogenous NEK7 in HEK293T/17 cells was sufficient, but could be enhanced by overexpression. Such overexpression of NEK7 did not enhance IL-1β cleavage in cells expressing NLRP3(R258W) (**Figure 2A**, lane 6 and lane 8).

Reconstituted NLRP3 in HEK293T/17 cells stimulated with nigericin has been published to cause IL-1β release detectable by ELISA (33). When we assayed IL-1β release by ELISA, we did observe a nigericin-dependent IL-1β ELISA signal. However, we also observed ASC independent IL-1β release (**Supplementary Figure 2A**), and notably HEK293T/17 cells do not express GSDMD that normally releases IL-1β from the cell. Addition of the NLRP3 agonist nigericin did not further increase cleavage of IL-1β in these cells by western blot (**Figure 2B**). These results suggest that this ELISA signal was a consequence of nigericin toxicity rather than NLRP3 signaling. Therefore, we did not continue to use nigericin or ELISA in our reconstitution experiments in HEK293T/17 cells.

### NLPR6 is expressed in intestinal epithelial cells and is prone to autoactivation

We used the same HEK293T/17expression system to study NLRP6, which was transfected together with ASC, caspase-1, and IL-1β. Remarkably, when NLRP6 was cotransfected with ASC and caspase-1, it resulted in pronounced cleavage of IL-1β (**Figure 2C**). Although caspase-11 has been published to promote NLRP6 signaling (35), when we added caspase-11 to the transfection, this did not enhance IL-1β cleavage (**Supplementary Figure 2B**). Similarly, NEK7 overexpression did not enhance NLRP6 signaling (**Supplementary Figure 2C**). Therefore, NLRP6 overexpression results in its autoactivation.

We next examined *Nlrp6* expression in several gene expression databases (BioGPS, ImmGen, and the Mouse Cell Atlas). *Nlrp6* appears to not be expressed in immune cells (**Supplementary Figures 2D–F**). qPCR results from cell lines also showed that *Nlrp6* was poorly expressed in macrophages (BMDMs, RAW264.7, J774A.1), and *NLRP6* was poorly expressed in human immune cell lines (HL-60, THP1, U937) and human epithelial cell lines (Caco2, T84) (**Supplementary Table 1**).

In contrast, results from tissue samples showed high *Nlrp6* expression in the intestine, but not in the spleen, bone marrow, or blood (**Figure 2D**). In support of this, a publicly available dataset from Reikvam et al. analyzing purified intestinal epithelial cells (IECs) showed that among mouse *Nlrp* genes, only *Nlrp6* was highly expressed in IECs (36) (**Figure 2E**). To confirm expression in IECs, we purified IECs from the small intestine and isolated RNA in comparison to RNA from bone marrow. *Nlrp6* was indeed expressed strongly in these IECs with negligible signal from bone marrow cells (**Figure 2F**). Interestingly, in the Reikvam et al. dataset *Nlrc4* and *Aim2* were also appreciably expressed (**Supplementary Figure 2G**), but at lower levels than *Nlrp6*. Overall, the NLRP6 inflammasome is expressed in IECs, but appears to not be expressed in immune cells.

### NLRP12 is an inflammasome that is less prone to autoactivation

**Figure 3:**
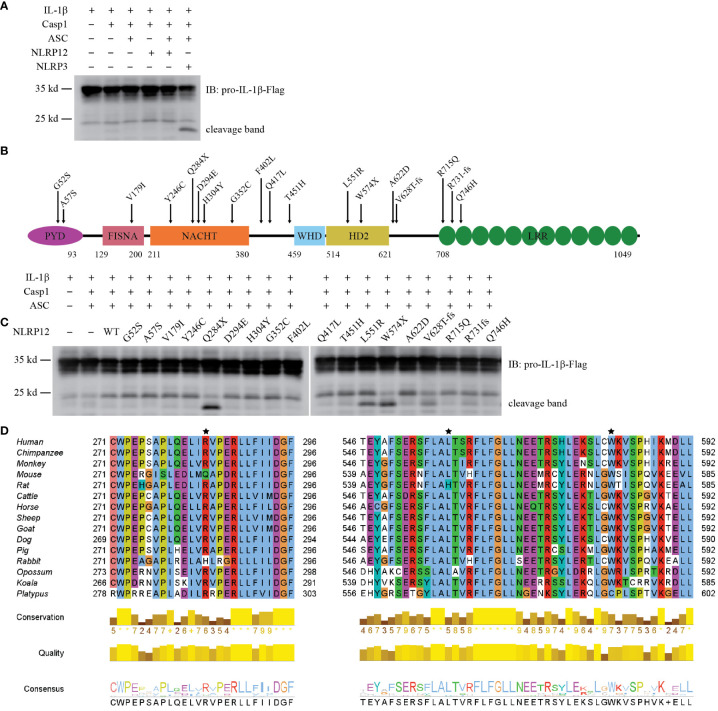
Reconstitution of NLRP12 inflammasome *in vitro*. **(A)** Reconstitution of NLRP12 inflammasome in HEK293T/17 cells with IL-1β, Caspase-1, ASC, NLRP12 (WT), and NLRP3 (as a positive control). Cleaved IL-1β was detected by western blot. **(B)** Schematic diagram of mouse NLRP12 mutants based on human NLRP12 mutants reported in human NLRP12-AID patients. **(C)** Reconstitution of NLRP12 inflammasome with IL-1β, caspase-1, ASC, NLRP12 (WT), and various NLRP12 mutants. Cleaved IL-1β was detected by western blot. **(D)** Alignment of NLRP12 protein sequence from indicated species. Asterisks indicate the mouse amino acids Gln^284^, Leu^551^ and Trp^574^ that correspond to residues mutated in NLRP12-AID patients (human Arg^284^, Leu^558^ and Trp^581^). Human, *Homo sapiens*; Chimpanzee, *Pan troglodytes*; Monkey, *Macaca mulatta*; Mouse, *Mus musculus*; Rat, *Rattus norvegicus*; Cattle, *Bos taurus*; Horse, *Equus caballus*; Sheep, *Ovis aries*; Goat, *Capra hircus*; Dog, *Canis lupus familiaris*; Pig, *Sus scrofa*; Rabbit, *Oryctolagus cuniculus*; Opossum, *Monodelphis domestica*; Koala, *Phascolarctos cinereus*; Platypus, *Ornithorhynchus anatinus*. All the results are representative of at least 3 independent experiments.

To study the function of NLRP12, we set up a similar activation assay in HEK293T/17 cells as used above. To our surprise, expression of full length NLRP12 did not induce IL-1β cleavage via ASC and caspase-1 (**Figure 3A**), suggesting that the protein is less prone to autoactivation compared to NLRP3 and NLRP6. The addition of NEK7 did not cause autoactivation of NLRP12 (**Supplementary Figure 3A**).

Some patients with periodic fever syndromes carry mutations in *NLRP12*. More than 20 *NLRP12* mutations have been reported (37–47). Many of these mutations have been reported to enhance NF-κB signaling (37, 40, 41, 43), however, whether they cause caspase-1-dependent cleavage of IL-1β has not been investigated. These patients carry NLRP12 mutations that cause single amino acid substitutions or truncations due to either premature stop codons or reading frame shifts (**Supplementary Figure 3B**). To test their effect on IL-1β cleavage, we generated the corresponding mutations in mouse NLRP12 (**Figure 3B**). All the mutants expressed well in HEK293T/17 cells, with similar levels of expression (**Supplementary Figure 3C**). Notably, though autoactivation was not observed in WT NLRP12-expressing cells, truncation mutants Q284X and W574X resulted in IL-1β cleavage (**Figure 3C**). The Q284X mutant, which only contains the PYD and FISNA domains, appeared to have the strongest autoactivation, as demonstrated by the robust cleavage of IL-1β. In comparison to the truncation mutants, the reading frameshift mutants V628T-fs and R731fs, which both truncate the LRR, resulted in modest IL-1β cleavage (**Figure 3C**). These results are consistent with the basic biochemistry common to the NLR family, which are often activated by truncation mutations. Most interestingly, the L551R mutant also resulted in strong IL-1β cleavage (**Figure 3C**); this was the only full-length protein for which we observed autoactivation.

NLRP12 protein sequence alignment analysis showed that amino acid residues 284, 551, and 574 were highly conserved among mammals (**Figure 3D**). The other patient-associated mutation sites were also conserved (**Supplementary Figure 3D**). Although the exact 3D structure of NLRP12 has not been reported, structural predictions by AlphaFold are available. Leu^551^ is buried within the protein (**Supplementary Figure 3E**), suggesting that an arginine substitution could be detrimental.

These results support the conclusion that NLRP12 forms an inflammasome whose activation is strictly regulated, but can be activated by certain mutations.

### NLRP3 and NLRP12 have distinct expression profiles in myeloid cell subtypes

**Figure 4:**
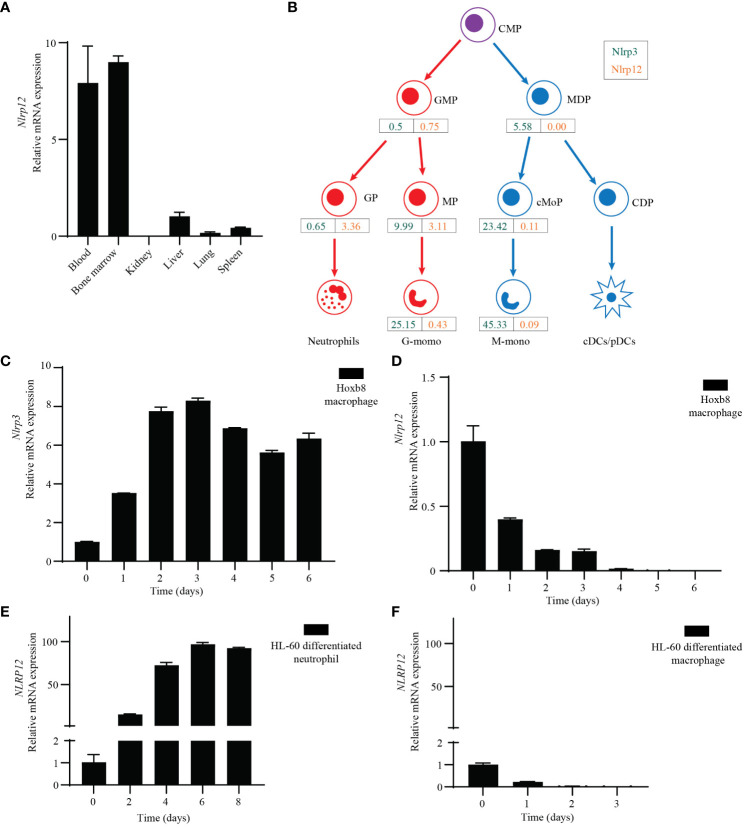
Specific expression profile of NLRP12 and NLRP3. **(A)** qRT-PCR analysis of *Nlrp12* expression in indicated mouse tissues. **(B)** *Nlrp3* (green) and *Nlrp12* (orange) expression by hematopoietic progenitors and monocytes was assessed in a previously published RNAseq dataset. RPKM values shown are means of duplicate samples of cells pooled from 20 mice each. CMP, common myeloid progenitors; GMP, granulocyte-monocyte progenitors; MDP, monocyte-dendritic cell progenitors; GP, granulocyte-committed progenitors; MP and cMoP, monocyte-committed progenitors; CDP, common dendritic cell progenitors; G-mono, GMP-derived monocytes; M-mono, MDP-derived monocytes; cDC and pDC, conventional and plasmacytoid dendritic cells. **(C, D)** qRT-PCR analysis of mouse **(C)** *Nlrp3* or **(D)** *Nlrp12* expression in Hoxb8-transduced progenitors that were induced to macrophage differentiation by L929 medium. **(E, F)** qRT-PCR analysis of *NLRP12* expression during HL-60 cell was induced to neutrophil-like cell differentiation by DMSO (1.25%) plus ATRA (1 μM) treatment or **(F)** macrophage differentiation by PMA (20 nM) treatment. All the results are representative of at least 3 independent experiments.

It is well established that NLRP3 is highly expressed in monocytes and macrophages. However, the expression pattern of NLRP12 is less defined. ImmGen, BioGPS, and the Mouse Cell Atlas all indicate that *Nlrp12* is mainly expressed in granulocytes, especially neutrophils and eosinophils, but is poorly expressed in other immune cells, including monocytes, macrophages, and dendritic cells (**Supplementary Figures 4A–C**). We performed qPCR from mouse tissues and found that *Nlrp12* was highly expressed in bone marrow and white blood cells (**Figure 4A**). RNA-seq data (GEO: GSE88982) demonstrated that the expression divergence of *Nlrp3* and *Nlrp12* occurs during hematopoietic stem cell (HSCs) differentiation through the myeloid lineage. Monocyte-dendritic cell progenitor (MDP)-derived cells (including monocyte-committed progenitors [cMoP], and the monocytes [M-mono] that they produce) express very low *Nlrp12*. In contrast, *Nlrp12* expression increases as granulocyte-monocyte progenitors (GMP) differentiate to their lineage-committed progeny, granulocyte progenitors (GP), which produce neutrophils. The branch toward GMP-derived monocyte progenitors (MP), also expressed *Nlrp12*, however this expression diminished upon differentiation into monocytes (G-mono) (**Figure 4B**, **Supplementary Figure 4D**). In contrast, *Nlrp3* expression increases during monocyte differentiation from either GMPs or MDPs, but *Nlrp3* remains very low in GP (**Figure 4B**, **Supplementary Figure 4D**). There were higher levels in the MDP pathway compared to the GMP pathway at each differentiation stage, however, both have high expression so slightly higher expression may not have a functional role. Note that terminally differentiated neutrophils and eosinophils were not evaluated in this experiment. Taken together, the summation of data indicates that *Nlrp12* expression is highly specific to neutrophils and eosinophils.

To study NLRP3 and NLRP12 during differentiation of macrophages and neutrophils *in vitro*, we transduced ER-Hoxb8 into common myeloid progenitor cells to create immortalized progenitors (48). We subjected these progenitor cells to macrophage differentiation with L929-conditioned media that contains M-CSF, and analyzed *Nlrp3* and *Nlrp12* expression by qPCR. Consistent with the above results, *Nlrp3* expression increased markedly during macrophage differentiation (**Figure 4C**). On the contrary, the expression of *Nlrp12* dramatically decreased (**Figure 4D**).

We also investigated expression of human NLRP12 using the HL-60 cell line derived from human acute promyelocytic leukemia (49). These cells can differentiate into either neutrophil-like or macrophage-like cells after different stimulation. Upon treatment with dimethyl sulfoxide (DMSO) and all-trans retinoic acid (ATRA) for 6 days, HL-60 cells differentiate into neutrophil-like cells, whereas upon PMA treatment for 3 days, HL-60 cells differentiate into macrophages (50). qPCR results showed that *NLRP12* expression was significantly increased during neutrophil differentiation (**Figure 4E**), but was dramatically decreased during macrophage differentiation (**Figure 4F**). In contrast, *NLRP3* expression did not change a lot during differentiation (**Supplementary Figures 4E, F**). This agrees with the data from ImmGen, where *Nlrp3* is expressed in both macrophages and neutrophils (**Supplementary Figure 4G**).

Therefore, NLRP12 is specifically expressed in neutrophils and eosinophils, but is not detectable in macrophages. In contrast, NLRP3 is highly expressed in monocyte/macrophages, and has low but appreciable expression in neutrophils.

### Inverse toxicity of NLRP3 and NLRP12 to macrophages and neutrophils

**Figure 5:**
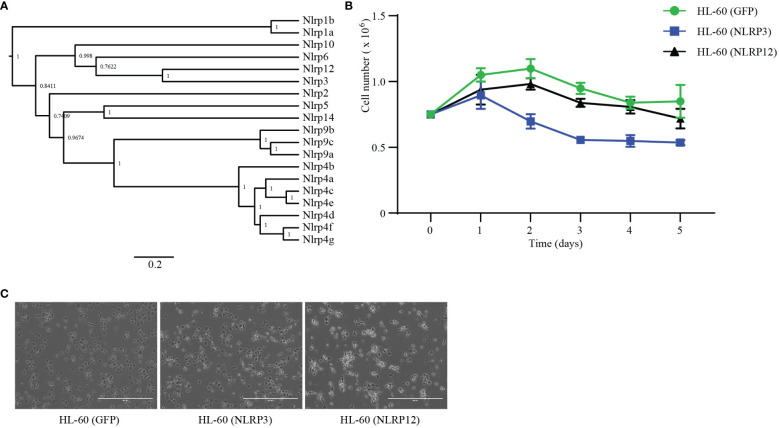
NLRP12 is toxic to macrophages. **(A)** Phylogenetic analysis of mouse NLRPs proteins. NLRP protein sequences were downloaded from NCBI. The sequences were analyzed by BEAST 1.10.4, which built the tree. **(B, C)** HL-60 cells were stably transduced with GFP, *NLRP3*, or *NLRP12*. **(B)** Stable cells were stimulated with DMSO (1.25%) plus ATRA (1 μM). The live cell numbers were counted at the indicated time point. **(C)** Stable cells were stimulated with PMA (20 nM) for 3 days and imaged by microcopy. Scale bars, 400μm. Results in **(B, C)** are representative of at least 3 independent experiments.

Molecular phylogeny analysis of all the mouse NLRP proteins showed that among NLRs, NLRP3 and NLRP12 are most closely related to each other (**Figure 5A**). Notably, NLRP12, NLRP3, NLRP6 and NLRP10, all of which were confirmed to result in IL-1β cleavage by the PYD domain scanning, clustered together. The AlphaFold prediction of NLRP12 was similar to NLRP3, except the PYD domain (**Supplementary Figure 5A**). In the NLRP12 prediction, the PYD blocked the LRR domain, which may explain why NLRP12 shows lower propensity to auto-activate upon overexpression.

To study the role of the expression patterns during the differentiation of macrophages and neutrophils, we constructed HL-60 stable cell lines that ectopically-expressed mouse *Nlrp3* or *Nlrp12*. We subjected these cells to neutrophil differentiation using DMSO and ATRA as before and assessed viability by counting the total number of non-adherent, neutrophil-like cells. NLRP12-overexpressing cells showed similar surviving cell numbers compared to the GFP-transfected control (**Figure 5B**). In contrast, over-expression of NLRP3 yielded fewer cells during the differentiation process beginning at day 2 (**Figure 5B**). On the other hand, during PMA-induced macrophage differentiation, we imaged each well to estimate the number of successfully differentiated macrophage-like cells that were adherent in the well. There were similar numbers of NLRP3-expressing cells compared to the GFP-expressing control cells (**Figure 5C**). In contrast, the number of NLRP12-expressing cells was markedly lower, and these cells had abnormal morphology (**Figure 5C**). Together, these results suggest that high levels of NLRP3 expression may be incompatible with neutrophil differentiation; conversely, NLRP12 expression may be incompatible with monocyte/macrophage differentiation.

### Cytosolic location of NLRP3 and NLRP12

**Figure 6:**
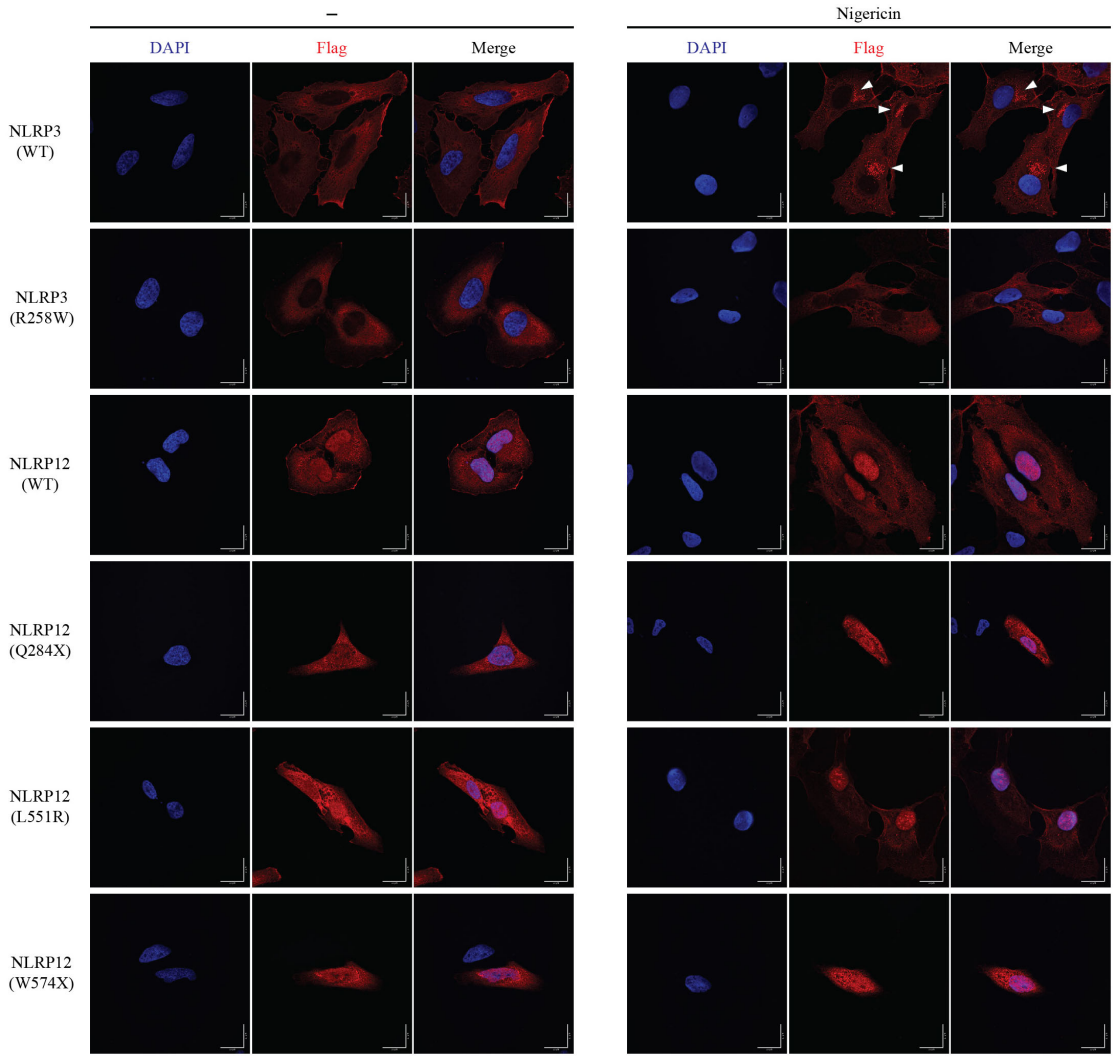
Subcellular location of NLRP3 and NLRP12. HeLa cells were stably transduced with mouse NLRP3 or NLRP12 and were stimulated with nigericin or not for 60 min. Immunostaining was performed for the Flag epitope tag and cells visualized by confocal microscopy. Triangles indicate NLRP3 foci. Scale bar, 20μm. All the images are representative of at least 3 independent experiments.

To explore the subcellular location of NLRP3 and NLRP12, we constructed Hela stable cell lines which ectopically-expressed the wild type and mutant of *Nlrp3* and *Nlrp12*. In the resting state, both NLRP3(WT) and NLRP3(R258W) were located in the cytosol. But after nigericin stimulation, NLRP3(WT) formed multiple foci in a peri-nuclear region, which matched the previous report that NLRP3 can be recruited to dispersed trans-Golgi network (dTGN) upon stimulation (51, 52). The expression of NLRP3(R258W) was weaker than wild type, which was coincident with the previous immunoblotting result (**Figure 2A**). Furthermore, the location was similar under resting state, which was also in the cytosol (**Figure 6**). To our surprise, the mutant NLRP3(R258W) did not form puncta after stimulation like NLRP3(WT). In addition to HeLa cells, we also transduced COS-1 cells with *Nlrp3*. NLRP3(WT) was also located in the cytoplasm in rest state, and formed numerous foci after nigericin stimulation (**Supplementary Figure 6B**). Interestingly, there were already some foci before stimulation, which might be due to some specific co-factors that only existed or were rich in COS-1 cells.

NLRP12(WT) was distributed not only in the cytosol but also was present in the nucleus (**Figure 6**). NLRP12(WT) did not form foci after nigericin treatment, which is different from NLRP3(WT). The cellular location of activating point mutation NLRP12(L551R) and NLRP12(F402L) were similar to NLRP12(WT), regardless of nigericin treatment. The truncation (NLRP12(Q284X) and NLRP12(W574X)) and frame shift (NLRP12 (V628T-fs) and NLRP12(R731-fs)) mutations were distributed throughout the cell, and did not response to nigericin stimulation (**Figure 6**, **Supplementary Figure 6A**), suggesting that the change in NLRP3 localization after nigericin treatment is specific to NLRP3. In COS-1 cells, NLRP12(WT) showed less nuclear location regardless of nigericin treatment, and again did not form foci after nigericin stimulation. The slight differences in nuclear exclusion may be due to cell line specific properties and suggest that the nuclear localization may not reflect an important aspect of NLRP12 function, but rather may reflect the ability of each cell line to exclude various proteins from the nucleus. The different localization patterns of NLRP3 and NLRP12 suggest that the activation mechanism of NLRP12 could be distinct from that of NLRP3.

## Discussion

Both inflammation and pyroptosis are double-edged swords, which are necessary to battle against pathogens but can also cause damage to tissues. Therefore, inflammasome activation should be under tight control. Here, we provide evidence that the NLRP proteins NLRP6 and NLRP12 can also form inflammasomes, similar to NLRP3, confirming prior reports that these are bona fide inflammasomes and could provide evidence against reports that they are not inflammasomes (53–56). It should be noted that our data carries caveats that are intrinsic to overexpression studies. These three inflammasomes are expressed in distinct cell types. The NLRP6 inflammasome is specifically expressed in IECs, while the NLRP12 inflammasome specifically exists in neutrophils and eosinophils. Meanwhile, NLRP3 is expressed in more cell types, including most myeloid cells such as macrophages, monocytes, DCs, mast cells, and neutrophils. Similarly, recent reports showed that NLRP10 also has restricted expression to either keratinocytes in the skin or intestinal epithelial cells of the distal colon (28, 29). Inflammasomes could also be inducible by specific cytokine stimulation in cell types that do not express them in the homeostatic state. All these cell types are exposed to infection by pathogens, but the different expression profiles of NLRP3, NLRP6, NLRP10, and NLRP12 probably reflects that each cell type has a distinct cell physiology that pathogens would evolve to target.

Each NLR could monitor a different aspect of cellular physiology or morphology that is unique to a specific cell type, and activate in response to a pathogen perturbing that aspect of cellular biology. To illustrate this concept, we will speculate on some possible examples of cell type-specific functions that could be monitored by these NLRs. Our data showing that NLRP6 is specifically expressed in IECs confirms several prior reports (57). IECs have vastly different morphology and immunologic capabilities compared to macrophages and neutrophils (58). IECs form a barrier and must absorb nutrients from the gut lumen (58, 59). To accomplish this, IECs have a large surface area created by microvilli, which are formed around densely polymerized actin structures (60). Microvilli are not present in immune cells. Speculatively, NLRP6 could function as a sensor (or a guard) (61) that monitors for the integrity of microvilli. Thus, if a pathogen disrupted the microvilli, this could cause NLRP6 to activate. Such a hypothetical case or another cell type specific property could explain why NLRP6 is highly autoactivate in HEK293T/17 cells – perhaps it is activating in response to the absence of microvilli. One would then expect NLRP6 to detect enteropathogenic *Escherichia coli* or *Citrobacter rodentium*, which remodel the microvilli; however, as these are successful in their native hosts, the pathogens would be expected to evade NLRP6 detection. Interestingly the two other organs that express NLRP6 are the liver and kidney, both of which contain epithelial cells with microvilli (60). Hepatocytes in the liver use microvilli to filter the blood, whereas brush border cells in the proximal tubule of the kidney use microvilli in ion exchange in the formation of urine. Speculatively, this type of cell type-specific structure could explain why only very specific cell types express NLRP6.

NLRP12 expression is highly restricted to neutrophils and eosinophils. Neutrophils share many properties with macrophages; however, our data suggest that NLRP12 might autoactivate in macrophages. There are many differences between macrophages and neutrophils. Neutrophils contain pre-formed granules that are essential for their antimicrobial function, which is also a characteristic of eosinophils (62). These granules must fuse with the phagosome in neutrophils, and also can be degranulated in neutrophils and eosinophils. Neutrophils and eosinophils also make considerably more reactive oxygen species using the NADPH oxidase than macrophages are capable of producing (62). We speculate that one of these properties could be a function that NLRP12 monitors, and activates only when the function is impeded by pathogen effectors.

Previous research reported that NLRP12 negatively regulates the NF-κB pathway via affecting the stability of NF-κB inducing kinase (NIK) (63). There were also reports showing that NLRP12 was a tumor suppressor gene. Allen. et al. found *Nlrp12^–/–^* mice were highly susceptible to colitis and colitis-associated colon cancer (64, 65). The authors attributed this to multiple signaling pathways, especially non-canonical NF-κB, all of which were negatively regulated by NLRP12. Udden et al. reported that *Nlrp12^–/–^* mice were highly susceptible to diethylnitrosamine-induced hepatocellular carcinoma (HCC) (66). The authors implicated NLRP12 as a negative regulator downregulation of JNK-dependent inflammation and proliferation of hepatocytes (66). Ulland et al. reported that in C57/B6J mouse, a missense mutation of *Nlrp12* (R1034K) caused neutrophil recruitment defect (67), which has been reported to affect tumorigenesis. Further investigation of these phenotypes could benefit from the knowledge that NLRP12 is an inflammasome, and that it is specifically expressed in neutrophils and eosinophils.

Our data support the conclusion of other prior studies that NLRP12 can form inflammasomes. Vladimer et al. found that NLRP12 recognized infection by a modified strain of *Yersinia pestis*, and mediated activation of caspase-1 and release of IL-1β and IL-18 (56). However, although these researchers and others had mentioned that *Nlrp12* was expressed more in neutrophils than macrophages (56, 68, 69), this conclusion has not been widely appreciated in subsequent papers. Here, we reinforce these conclusions by showing that NLRP12 is primarily expressed in neutrophils, and also in eosinophils. Recent work Recent work from Coombs et al. used PBMCs stimulated with LPS for 24 hours, which should not contain neutrophils or eosinophils (70). Other recent work from Sundaram et al. studied NLRP12 primarily in murine bone marrow-derived macrophages, a cell type that we found to express undetectable levels of *Nlrp12* message (71). The researchers reported NLRP12 activated in response to heme and mediated PANoptosis with ASC, caspase-8, and RIPK3 (71). In that paper, the researchers found the expression of *Nlrp12* increased more 10 times only after 36 hours of LPS or Pam_3_CSK_4_ stimulation (71), which is longer than typical priming treatments used during *in vitro* studies. Similarly, the pyrin inflammasome is normally expressed by neutrophils, and to a lesser extent monocytes, but not by macrophages. However, 24 hours of TLR or cytokine stimulation will modestly induce pyrin expression in monocytes or macrophages (72, 73). Thus, prolonged stimulation in macrophages can induce expression of the NLRP12 or pyrin inflammasomes, which could be relevant to *in vivo* infections where pathogens typically linger for many days.

Clinical researchers have reported an autoinflammatory disease caused by NLRP12 mutation (called NLRP12-AID). The patients typically presented with periodic fever, urticaria-like rash, arthralgia/arthritis, myalgia, and lymphadenopathy (41). Among the patients, most developed the disease in childhood. Through exon sequence or other methods, the researchers have reported more than 20 NLRP12 variants, including point mutation, truncation, and reading frame shifts. Some researchers have found increased inflammatory cytokine secretion, including IL-1β, TNFα, and IFN-γ in some patients’ serum (37, 39, 43). The patients’ PBMC cells were more sensitive to LPS stimulation than control cells from health people. The authors attributed these to activation of the NF-κB pathway; a few mutants tested with NF-κB responsive luciferase showed a loss of the inhibitory efforts of NLRP12 (40, 41), but many mutants did not show this activity. A recent report suggested that NLRP12 is not an inflammasome, but instead is a tonic inhibitor of NLRP3, and that the patient-associated mutations did not activate ASC signaling, including the human V635T frame shift that deletes the LRR domain (70). In direct contrast to this, we now show that several mutations in NLRP12-AID patients (including the mouse equivalent of V635Tfs) cause spontaneous activation of caspase-1. However, other NLRP12 mutations did not cause caspase-1 activation. We speculate that some of these patient mutations could cause spontaneous activity in neutrophils, and that there may be neutrophil specific factors that are missing from HEK293T/17 cells that cause some of these mutations to fail to autoactivate in our studies. This could be analogous to how NLRP3 needs NEK7; NLRP12 may require a co-factor that is only expressed in neutrophils. Alternately, it may be that some NLRP12 amino acid substitutions are not actually causative of the patient’s disease. The F402L mutation has been reported by several clinical papers to be present in patients with autoinflammatory syndromes, and F402 is highly conserved among species (**Supplementary Figure 3D**). However, F402L is a common polymorphism found in humans who do not have autoinflammatory disease, and thus this mutation has been proposed to not be causative of autoinflammatory disease (38), a conclusion that is supported by our data where F402L is not an activating mutation.

IL-1β blockade has been used in 2 NLRP12-AID patients – Anakinra therapy achieved a marked clinical improvement at the first 2 weeks, secretion of IL-1β by peripheral blood mononuclear cells (PBMCs) decreased significantly within 2 months of treatment (74). However, over time the efficacy was lost and treatment was discontinued after 14 months (74). It may be that these patients have symptoms driven by the combined effects of IL-1β and IL-18. In this regard, NLRC4 auto-activating mutations received therapeutic benefit from IL-18 blockade (75). However, pyroptosis also releases multiple cytosolic molecules that are inflammatory, and patients might additionally need to be treated with drugs that inhibit GSDMD (76).

## Data availability statement

The datasets presented in this study can be found in online repositories. The names of the repository/repositories and accession number(s) can be found in the article/**Supplementary Material**.

## Ethics statement

Ethical approval was not required for the studies on humans in accordance with the local legislation and institutional requirements because only commercially available established cell lines were used. Animal study protocols were approved by the Institutional Animal Care and Use Committee (IACUC) at Duke University School of Medicine (protocols A043-20-02 approved February 27, 2020, and A018-23-01 approved February 2, 2023) and met guidelines of the US National Institutes of Health for the humane care of animals.

## Author contributions

BW: Conceptualization, Data curation, Formal analysis, Investigation, Methodology, Project administration, Resources, Software, Validation, Visualization, Writing – original draft, Writing – review & editing. ZB: Methodology, Software, Writing – review & editing. KN: Methodology, Writing – review & editing. HG: Funding acquisition, Methodology, Writing – review & editing. EM: Funding acquisition, Methodology, Project administration, Supervision, Writing – review & editing.

## Supplementary material

Supplementary Figure 1 The expression of CARD and PYD domain fusion proteins. **(A)** The diagram of NLR family proteins. **(B, C)** The diagram of NLRC4 **(B)** and NLRP3 **(C)** activate downstream Caspase-1. **(D, E)** The diagram of CARD domain **(D)** and PYD domain **(E)** fusion proteins activate downstream Caspase-1. **(E)** The expression of mouse CARD domain fusion proteins. The CARD domain from *Apaf*, *Aire*, *Ciita*, *Nlrc3*, *Nlrc4*, and N-terminal domain of *Nlrx1* were subcloned and expressed together with tandem DmrB domain as fusion proteins. **(F)** The expression of mouse PYD domain fusion proteins. The PYD domain from *Nlrp2, Nlrp3, Nlrp4a*, *Nlrp4b*, *Nlrp4c*, *Nlrp4d*, *Nlrp4e*, *Nlrp4f*, *Nlrp4g, Nlrp6*, *Nlrp9a*, *Nlrp9b*, *Nlrp9c, Nlrp10*, *Nlrp12*, and *Nlrp14* were subcloned and expressed together with tandem DmrB domain as fusion proteins. **(G)** The expression of human PYD domain fusion proteins. The PYD domain from *NLRP1*, *NLRP2*, *NLRP3, NLRP4*, *NLRP5*, *NLRP6*, *NLRP7*, *NLRP8*, *NLRP9*, *NLRP10*, *NLRP11*, *NLRP12*, *NLRP13*, and *NLRP14* were subcloned and expressed together with tandem DmrB domain as fusion proteins. The HEK293T/17 cells were transiently transfected with indicated plasmid for 24h, and lysates were subjected to immunoblotting with indicated antibody. All the blotting results are representative of at least 3 independent experiments.

Supplementary Figure 2 Reconstitution of inflammasome *in vitro*. **(A)** NLRP3, NLRP6 and NLRP12 inflammasome were reconstituted in HEK293T/17 cells using the indicated plasmids with or without nigericin treatment. IL-1β concentration in cell culture medium was assayed by ELISA. **(B, C)** Reconstitution of NLRP6 inflammasome with *Casp11* **(B)** and NEK7 **(C)** with indicated immunoblots. **(D–F)** The expression of mouse *Nlrp6* from BioGPS **(D)**, ImmGen **(E)** and the Mouse Cell Altas **(F)**. The results were downloaded from BioGPS, ImmGen (RNA-seq), and the Mouse Cell Altas websites. **(G)** The expression of *Aim2*, *Pyrin*, and *Nlrc4* in IECs. The original data is downloaded from NCBI (GDS3921). The results were normalized by albumin (*Alb*). The expression of Ald was taken as 1, the ration of Nlrps to Alb represented the expression of Nlrps. The results in **(A–C)** are representative of at least 3 independent experiments.

Supplementary Figure 3 The character of NLRP12. **(A)** Reconstitution of NLRP12 inflammasome with IL-1β, Caspase-1, ASC, NEK7, NLRP12 (WT) and NLRP3 (as a positive control) with indicated immunoblots. **(B)** Schematic diagram of human NLRP12 mutants, which have been reported in human NLRP12-AID patients by clinical researchers before. **(C)** The expression of various NLRP12 mutants and wild type in HEK293T/17 cells. **(D)** Alignment of NLRP12 from indicated species. The asterisk indicated the mutation sites in panel **(B)** and **Figure 3B**. **(E)** The predicted structure of mouse NLRP12 by AlphaFold. The date file was downloaded from AlphaFold protein structure database, and analyzed by iCn3D. (https://www.ncbi.nlm.nih.gov/Structure/icn3d/full.html). Leu^551^ was labeled yellow. The blotting results in **(A, C)** are representative of at least 3 independent experiments.

Supplementary Figure 4 Specific expression of NLRP12 and NLRP3. **(A-C)** The expression of *Nlrp12* from ImmGen **(A)**, BioGPS **(B)**, and the Mouse Cell Altas **(C)**. The results were downloaded from BioGPS, ImmGen (RNA-seq) and the Mouse Cell Altas websites. **(D)** The expression of *Nlrp3* and *Nlrp12* from **Figure 4B** depicted as a bar graph. (E, F) qRT-PCR analysis of *NLRP3* expression. The samples in **Figure 4E** **(E)** and **Figure 4F** **(F)** were assayed for *NLRP3* expression. **(G)** The expression of *Nlrp3* from ImmGen. Data from lung basophils from mice infected with *Nippostrongylus brasiliensis* were excluded from the graph as these had extraordinarily high expression of *Nlrp3* that obscured visualization of the basal expression in other cell types. The results in **(D–F)** are representative of at least 3 independent experiments.

Supplementary Figure 5 Alignment the NLRP12 structure with NLRP3 structure. The structure files of mouse NLRP3 (green) and NLRP12 (magenta) were downloaded from AlphaFold protein structure database and aligned by PyMOL.

Supplementary Figure 6 The subcellular location of NLRP3 and NLRP12. **(A)** The subcellular location of NLRP12(F402L, V628T-fs, R731-fs) in HeLa stable cells. The HeLa cells stably expressing NLRP12 were stimulated with nigericin or not. Immunostaining was performed for the Flag epitope tag and cells visualized by confocal microscopy. Scale bar, 20μm. **(B)** The subcellular location of NLRP3 and NLRP12 in COS-1 stable cells. The COS-1 cells stably expressing mouse NLRP3 and NLRP12 were stimulated with nigericin or not. Immunostaining was performed for the Flag epitope tag and cells visualized by confocal microscopy. Scale bar, 20μm. Triangles indicate the NLRP3 foci. All the images are representative of at least 3 independent experiments.

Supplementary Table 1 Expression of *Nlrp6* and *NLRP6* in various cell lines. qRT-PCR analysis of *Nlrp6* and *NLRP6* expression in indicated mouse and human cell1lines. The table showed the raw CT value. All the results are representative of at least 3 independent experiments.
