# Supplementary figures and images for: NLRP3, NLRP6, and NLRP12 are inflammasomes with distinct expression patterns
Source: Front Immunol. 2024 Jul 15;15:1418290. doi: 10.3389/fimmu.2024.1418290 (PMC11284034; doi:10.3389/fimmu.2024.1418290)

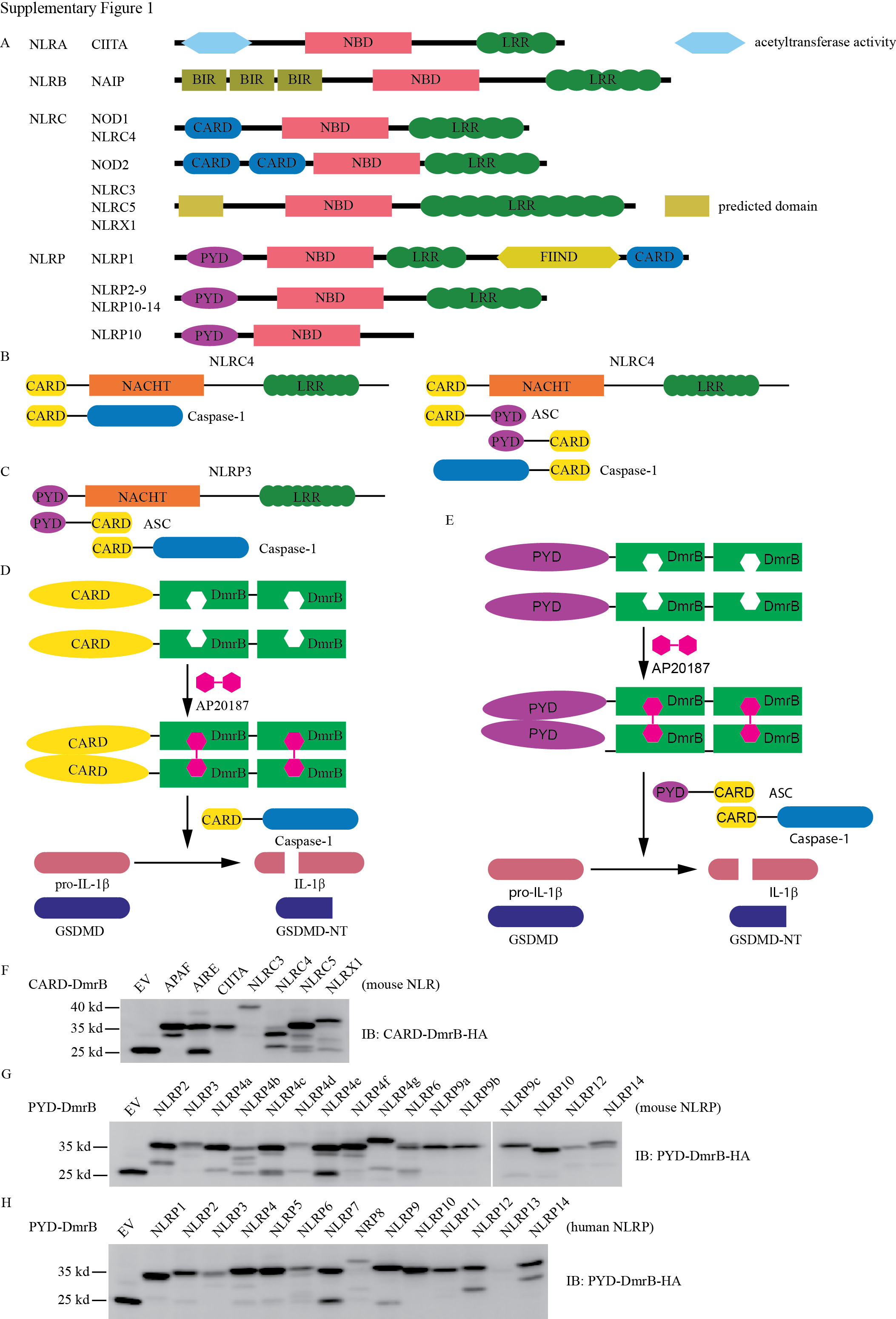

Supplement: Supplementary Figure 1 — The expression of CARD and PYD domain fusion proteins. (A) The diagram of NLR family proteins. (B, C) The diagram of NLRC4 (B) and NLRP3 (C) activate downstream Caspase-1. (D, E) The diagram of CARD domain (D) and PYD domain (E) fusion proteins activate downstream Caspase-1. (E) The expression of mouse CARD domain fusion proteins. The CARD domain from Apaf, Aire, Ciita, Nlrc3, Nlrc4, and N-terminal domain of Nlrx1 were subcloned and expressed together with tandem DmrB domain as fusion proteins. (F) The expression of mouse PYD domain fusion proteins. The PYD domain from Nlrp2, Nlrp3, Nlrp4a, Nlrp4b, Nlrp4c, Nlrp4d, Nlrp4e, Nlrp4f, Nlrp4g, Nlrp6, Nlrp9a, Nlrp9b, Nlrp9c, Nlrp10, Nlrp12, and Nlrp14 were subcloned and expressed together with tandem DmrB domain as fusion proteins. (G) The expression of human PYD domain fusion proteins. The PYD domain from NLRP1, NLRP2, NLRP3, NLRP4, NLRP5, NLRP6, NLRP7, NLRP8, NLRP9, NLRP10, NLRP11, NLRP12, NLRP13, and NLRP14 were subcloned and expressed together with tandem DmrB domain as fusion proteins. The HEK293T/17 cells were transiently transfected with indicated plasmid for 24h, and lysates were subjected to immunoblotting with indicated antibody. All the blotting results are representative of at least 3 independent experiments. [file Image_1.jpeg]

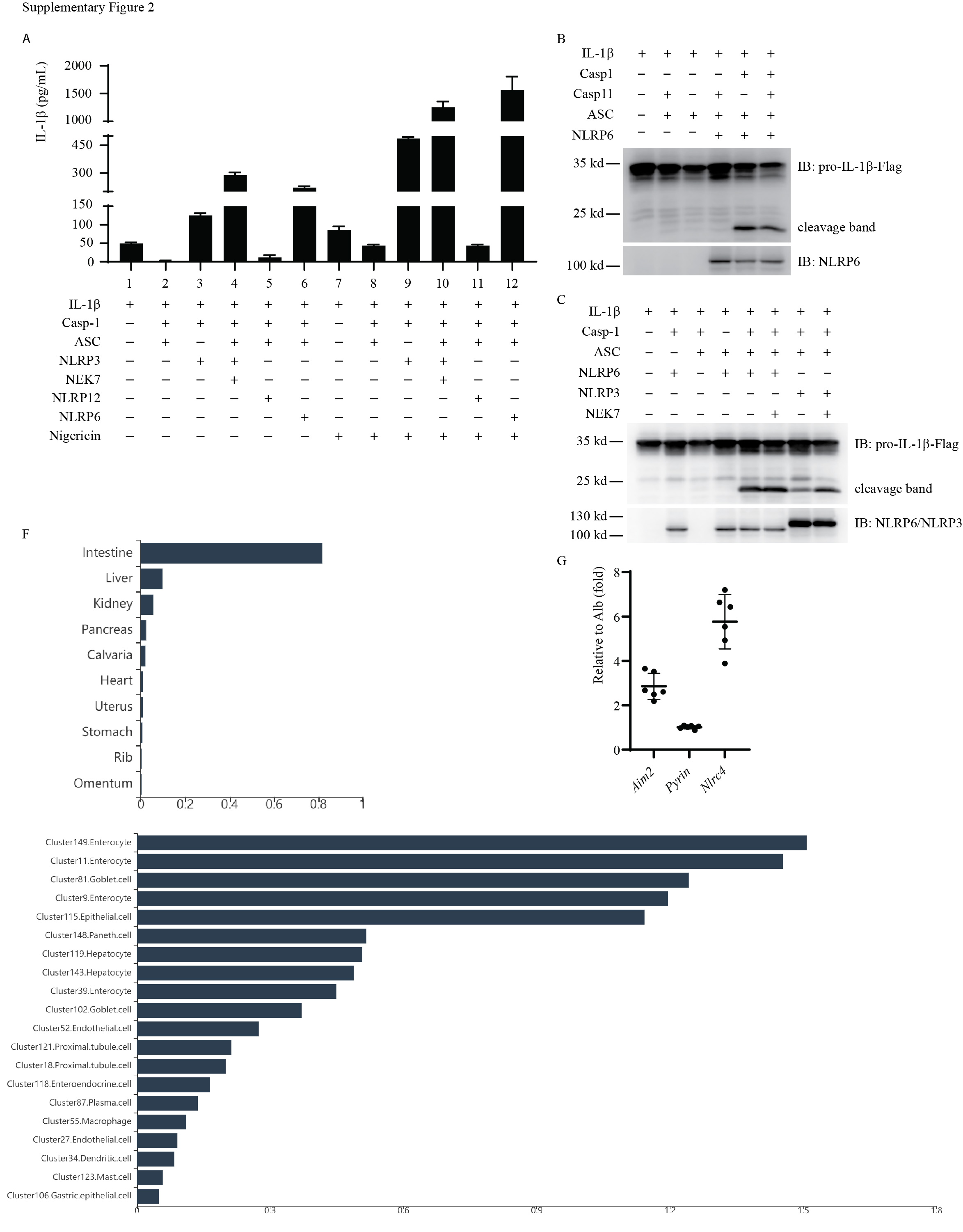

Supplement: Supplementary Figure 2 — Reconstitution of inflammasome in vitro. (A) NLRP3, NLRP6 and NLRP12 inflammasome were reconstituted in HEK293T/17 cells using the indicated plasmids with or without nigericin treatment. IL-1β concentration in cell culture medium was assayed by ELISA. (B, C) Reconstitution of NLRP6 inflammasome with Casp11 (B) and NEK7 (C) with indicated immunoblots. (D–F) The expression of mouse Nlrp6 from BioGPS (D), ImmGen (E) and the Mouse Cell Altas (F). The results were downloaded from BioGPS, ImmGen (RNA-seq), and the Mouse Cell Altas websites. (G) The expression of Aim2, Pyrin, and Nlrc4 in IECs. The original data is downloaded from NCBI (GDS3921). The results were normalized by albumin (Alb). The expression of Ald was taken as 1, the ration of Nlrps to Alb represented the expression of Nlrps. The results in (A–C) are representative of at least 3 independent experiments. [file Image_2.jpeg]

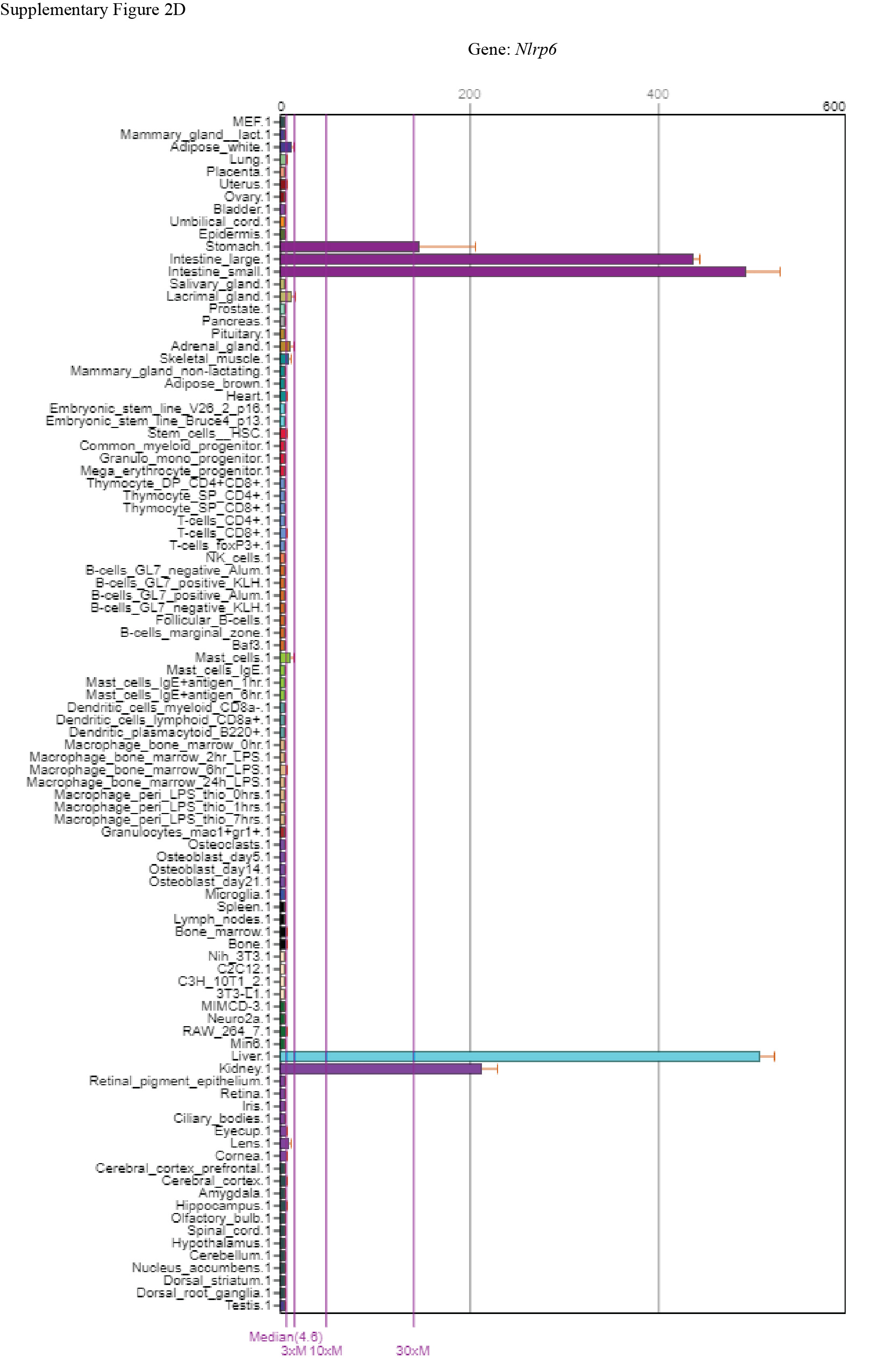

Supplement: Supplementary Figure 3 — The character of NLRP12. (A) Reconstitution of NLRP12 inflammasome with IL-1β, Caspase-1, ASC, NEK7, NLRP12 (WT) and NLRP3 (as a positive control) with indicated immunoblots. (B) Schematic diagram of human NLRP12 mutants, which have been reported in human NLRP12-AID patients by clinical researchers before. (C) The expression of various NLRP12 mutants and wild type in HEK293T/17 cells. (D) Alignment of NLRP12 from indicated species. The asterisk indicated the mutation sites in panel (B) and Figure 3B . (E) The predicted structure of mouse NLRP12 by AlphaFold. The date file was downloaded from AlphaFold protein structure database, and analyzed by iCn3D. (https://www.ncbi.nlm.nih.gov/Structure/icn3d/full.html). Leu551 was labeled yellow. The blotting results in (A, C) are representative of at least 3 independent experiments. [file Image_3.jpeg]

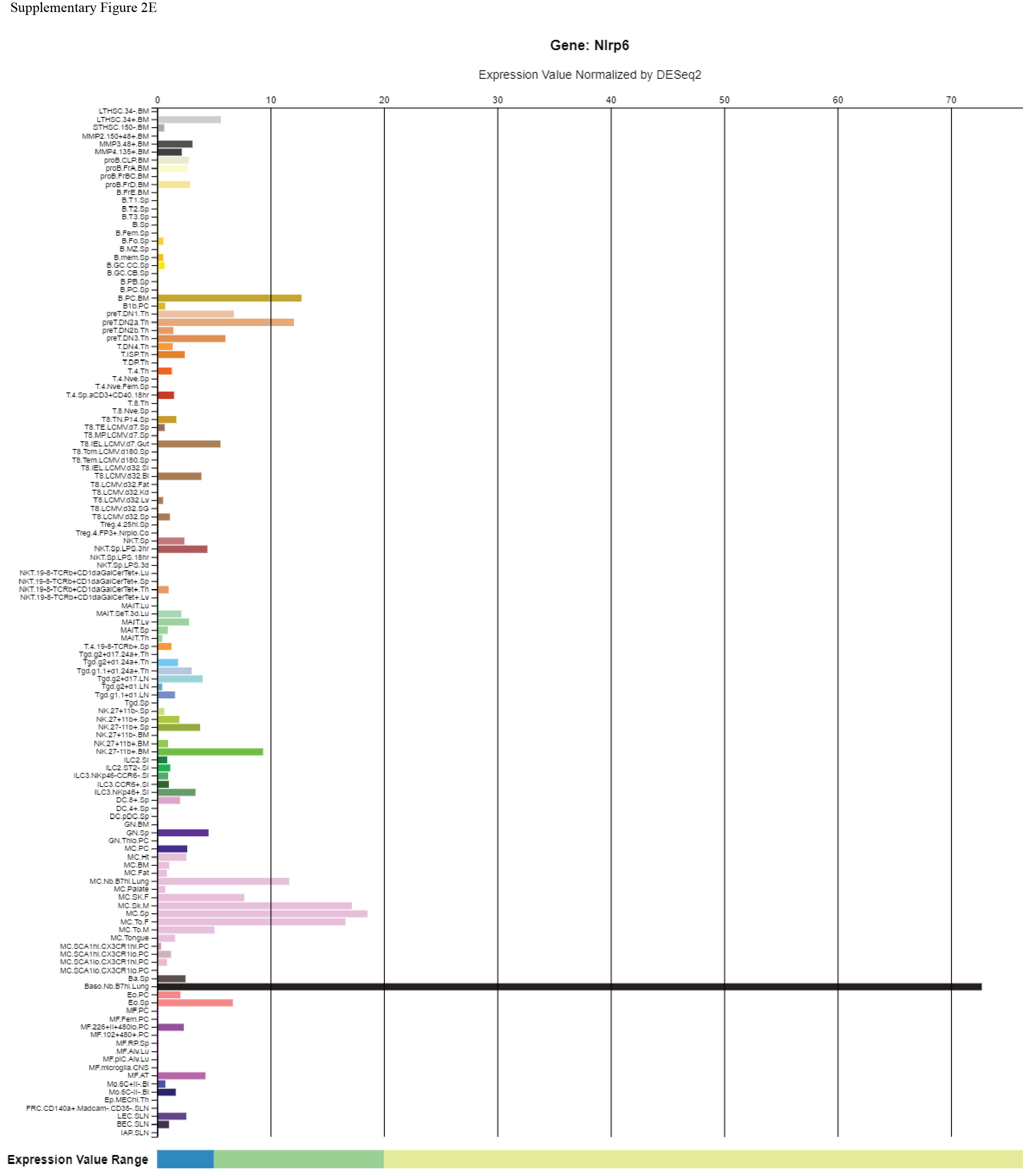

Supplement: Supplementary Figure 4 — Specific expression of NLRP12 and NLRP3. (A-C) The expression of Nlrp12 from ImmGen (A), BioGPS (B), and the Mouse Cell Altas (C). The results were downloaded from BioGPS, ImmGen (RNA-seq) and the Mouse Cell Altas websites. (D) The expression of Nlrp3 and Nlrp12 from Figure 4B depicted as a bar graph. (E, F) qRT-PCR analysis of NLRP3 expression. The samples in Figure 4E (E) and Figure 4F (F) were assayed for NLRP3 expression. (G) The expression of Nlrp3 from ImmGen. Data from lung basophils from mice infected with Nippostrongylus brasiliensis were excluded from the graph as these had extraordinarily high expression of Nlrp3 that obscured visualization of the basal expression in other cell types. The results in (D–F) are representative of at least 3 independent experiments. [file Image_4.jpeg]

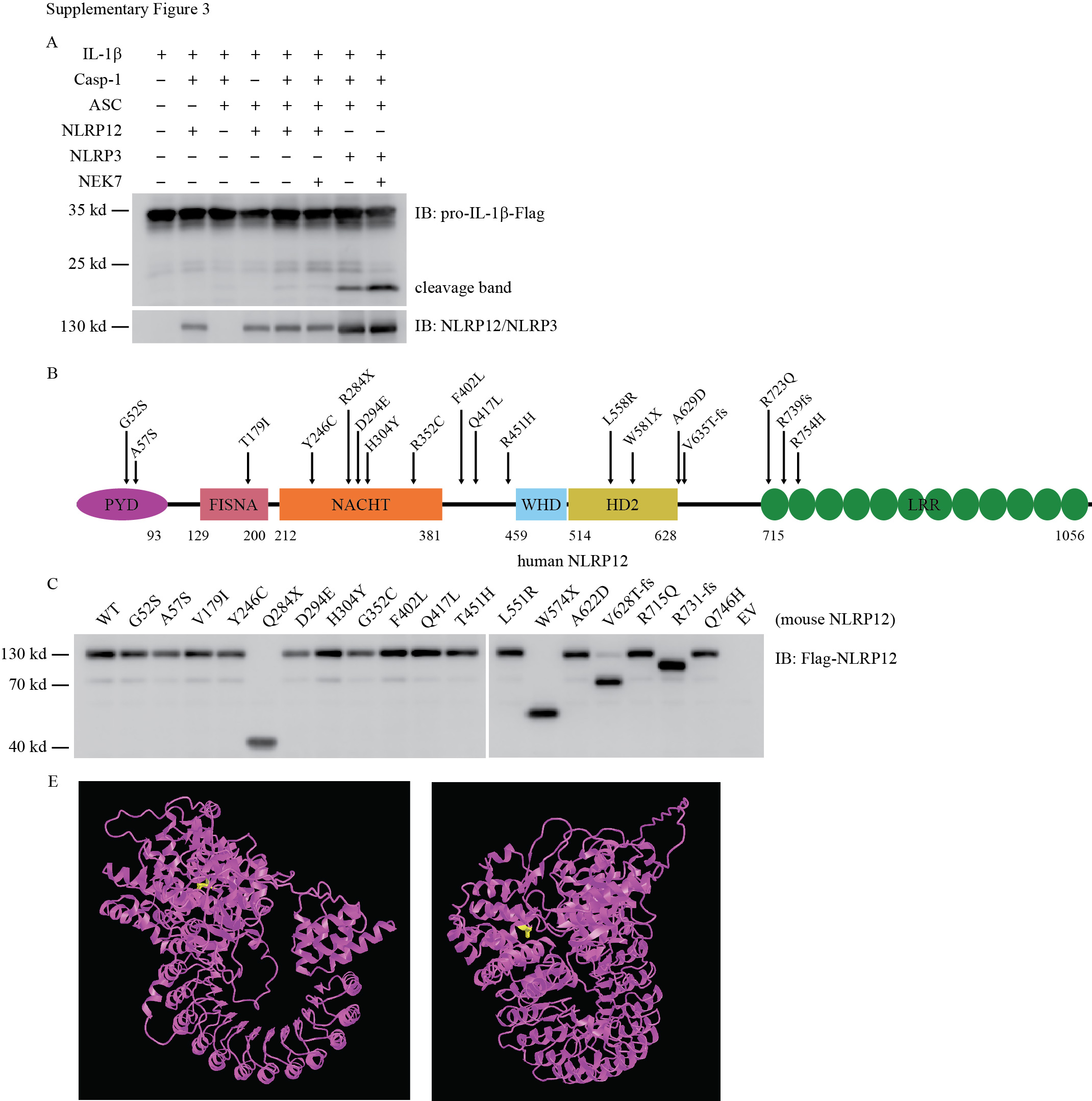

Supplement: Supplementary Figure 5 — Alignment the NLRP12 structure with NLRP3 structure. The structure files of mouse NLRP3 (green) and NLRP12 (magenta) were downloaded from AlphaFold protein structure database and aligned by PyMOL. [file Image_5.jpeg]

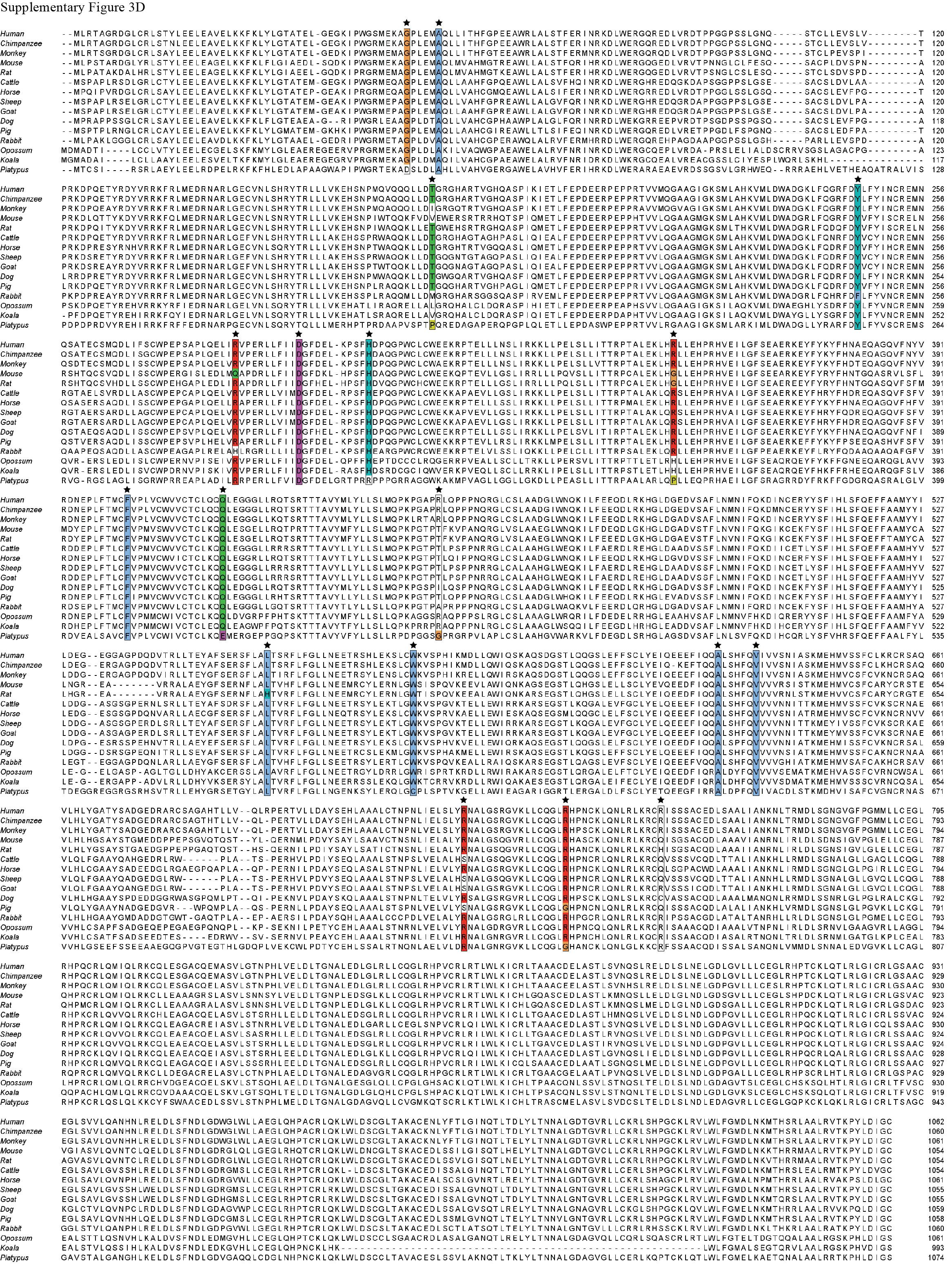

Supplement: Supplementary Figure 6 — The subcellular location of NLRP3 and NLRP12. (A) The subcellular location of NLRP12(F402L, V628T-fs, R731-fs) in HeLa stable cells. The HeLa cells stably expressing NLRP12 were stimulated with nigericin or not. Immunostaining was performed for the Flag epitope tag and cells visualized by confocal microscopy. Scale bar, 20μm. (B) The subcellular location of NLRP3 and NLRP12 in COS-1 stable cells. The COS-1 cells stably expressing mouse NLRP3 and NLRP12 were stimulated with nigericin or not. Immunostaining was performed for the Flag epitope tag and cells visualized by confocal microscopy. Scale bar, 20μm. Triangles indicate the NLRP3 foci. All the images are representative of at least 3 independent experiments. [file Image_6.jpeg]

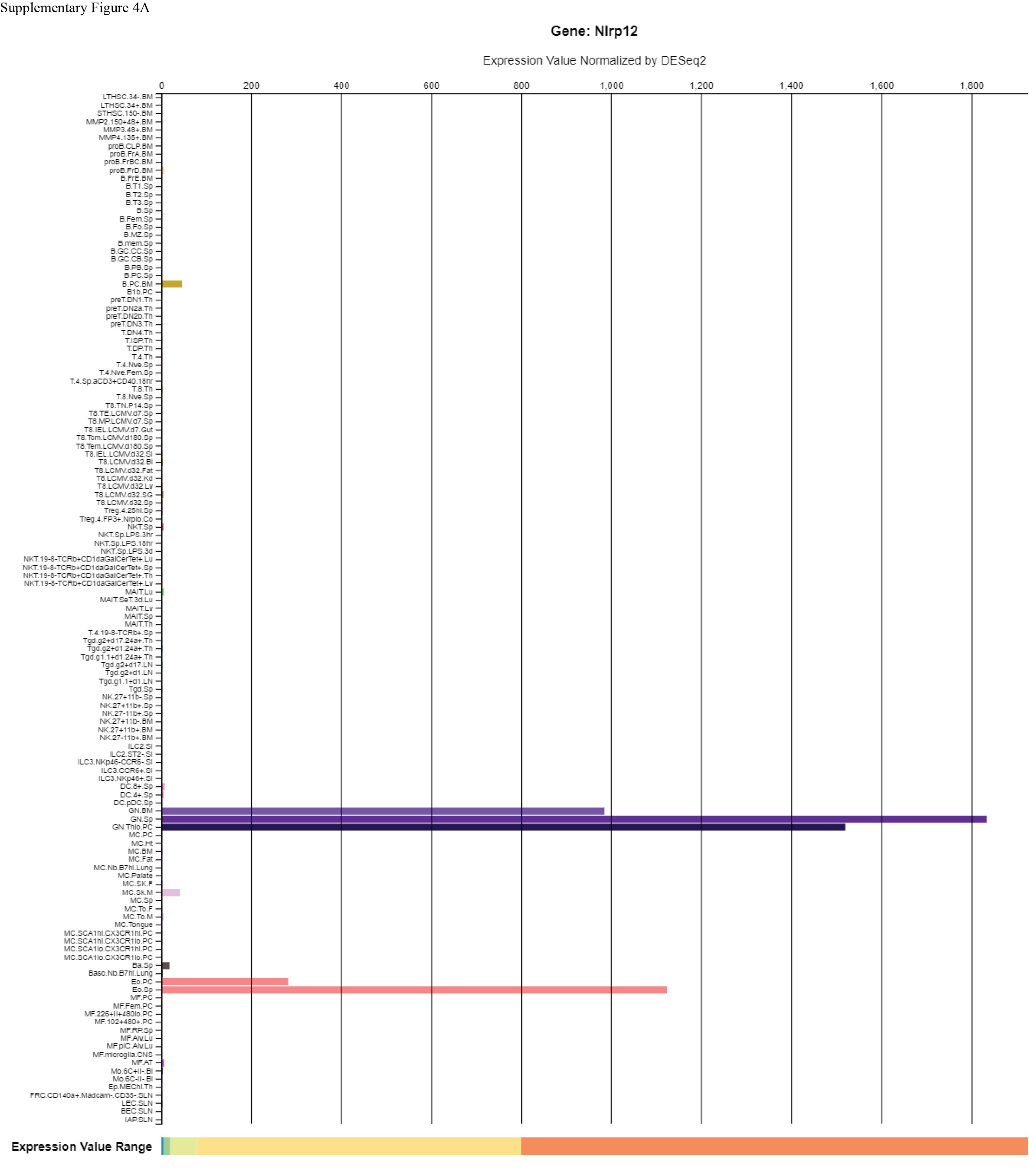

Supplement: Supplementary file 7 [file Image_7.jpeg]

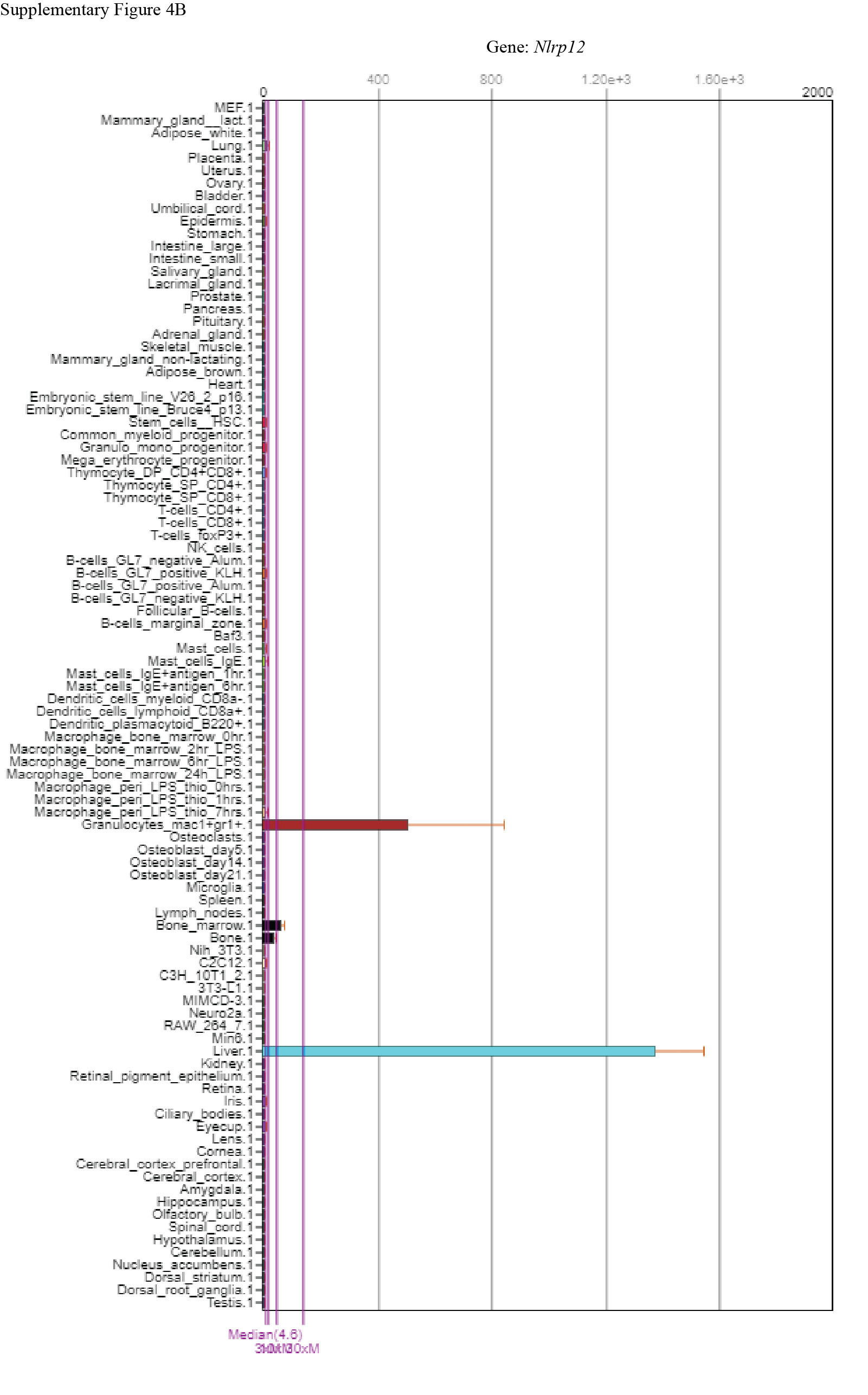

Supplement: Supplementary file 8 [file Image_8.jpeg]

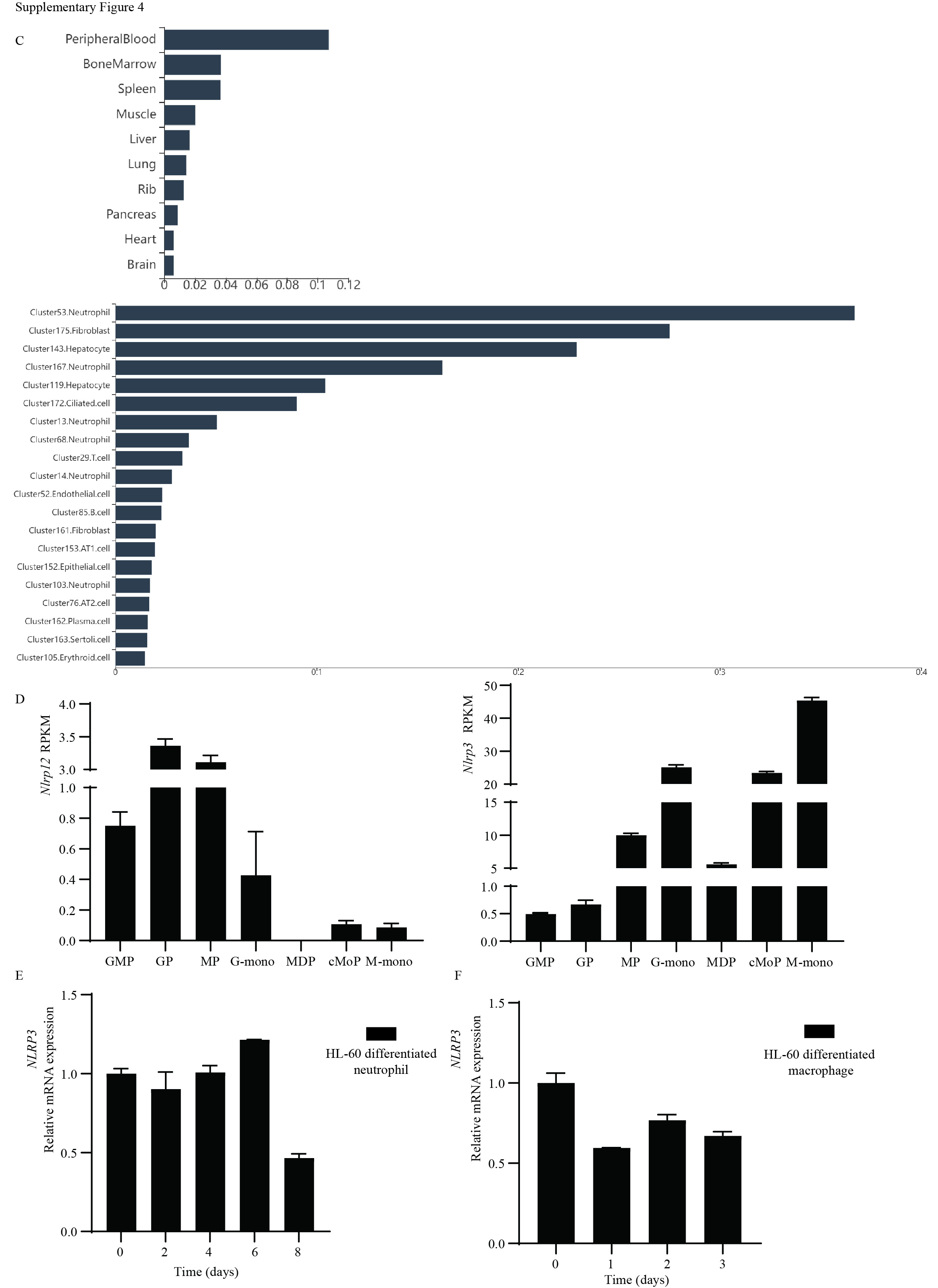

Supplement: Supplementary file 9 [file Image_9.jpeg]

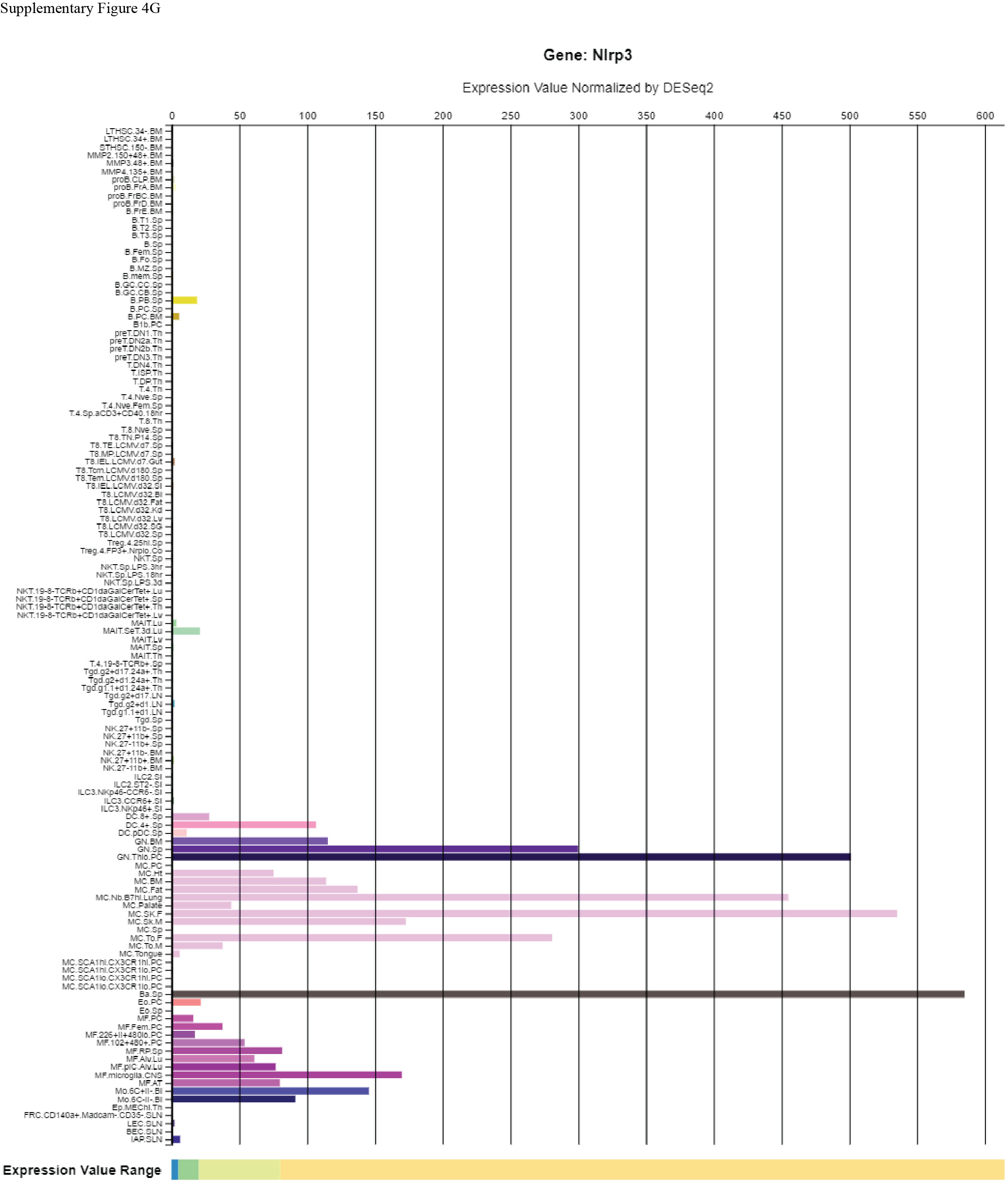

Supplement: Supplementary file 10 [file Image_10.jpeg]

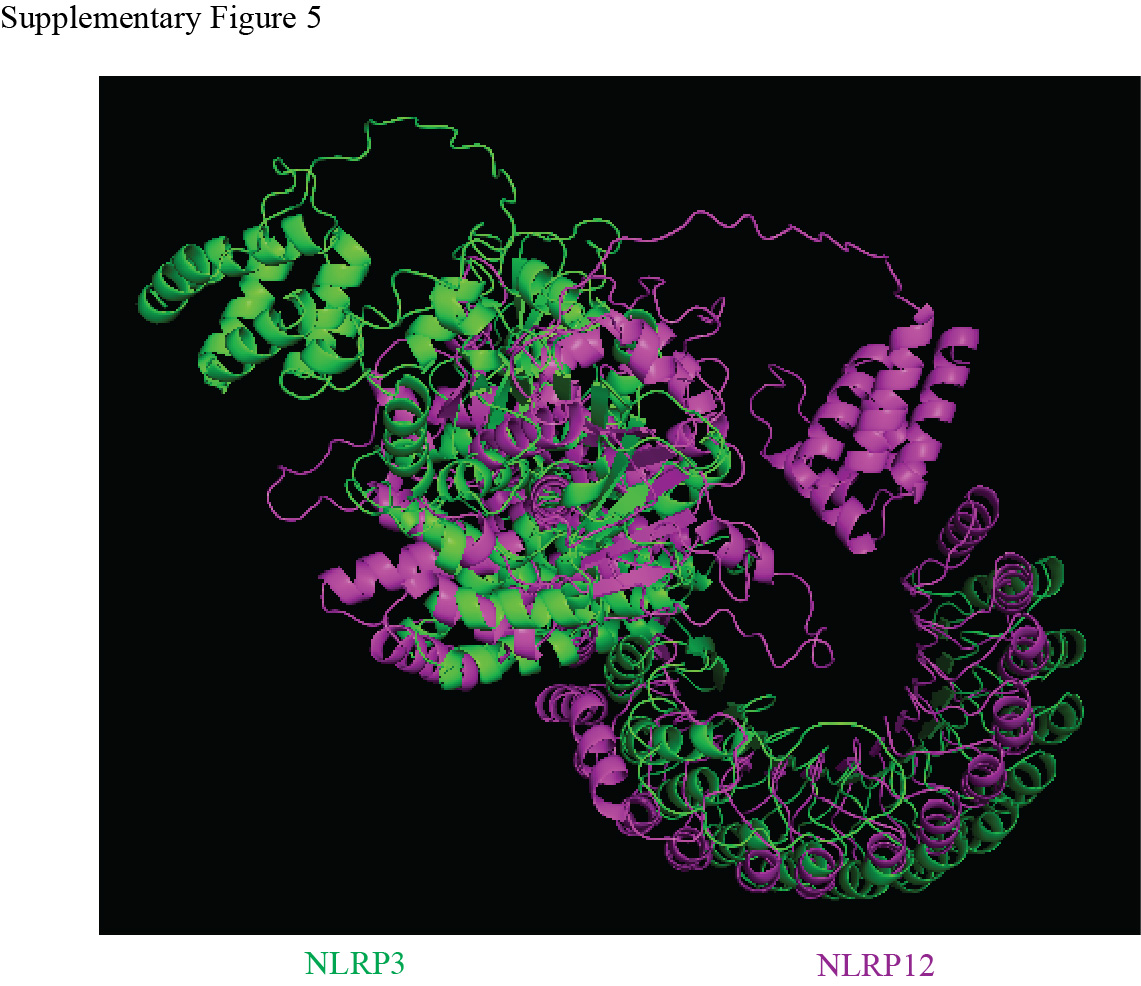

Supplement: Supplementary file 11 [file Image_11.jpeg]

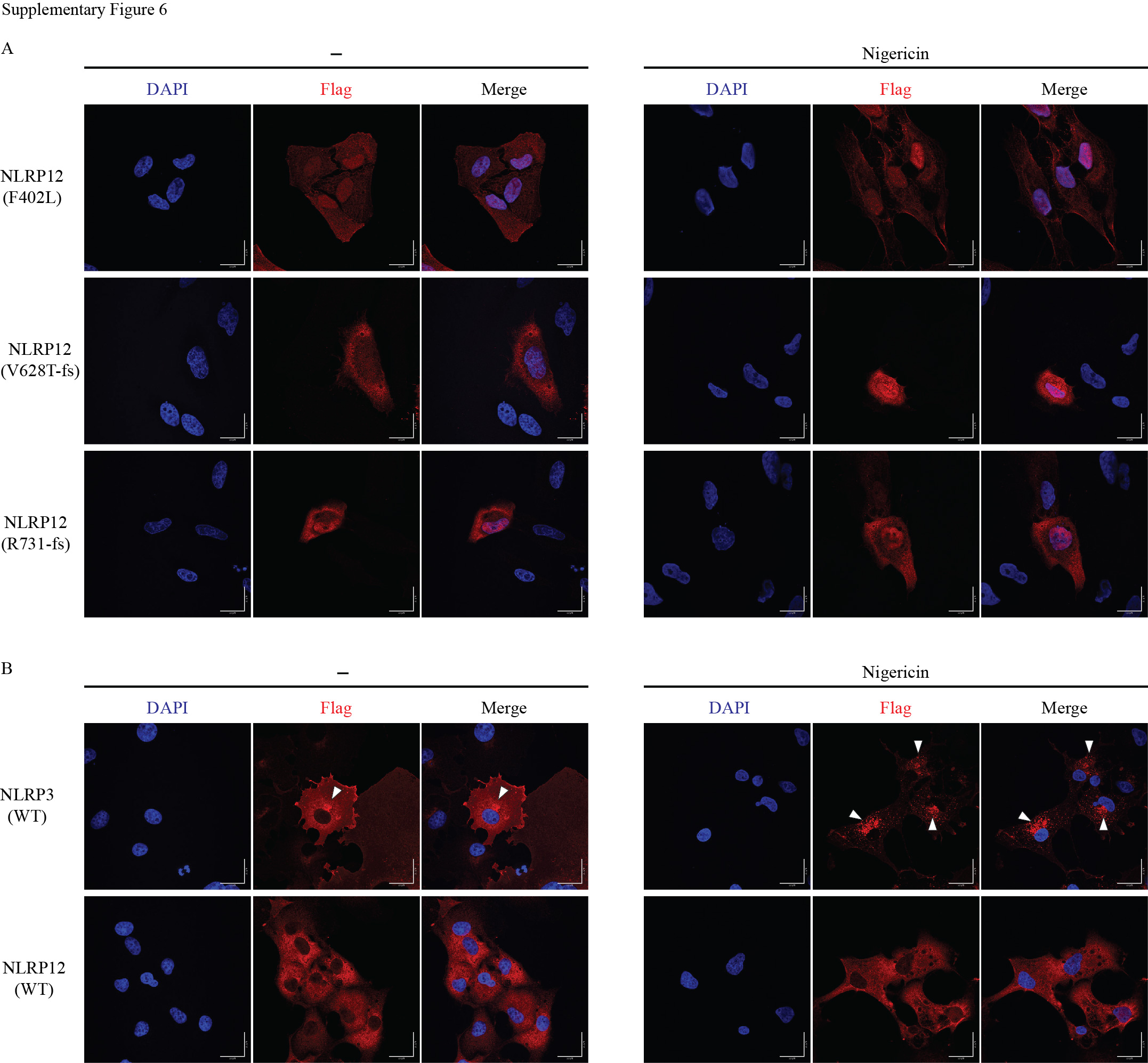

Supplement: Supplementary file 12 [file Image_12.jpeg]
