# Supplementary material for: NLRP3, NLRP6, and NLRP12 are inflammasomes with distinct expression patterns
Source: Front Immunol. 2024 Jul 15;15:1418290. doi: 10.3389/fimmu.2024.1418290 (PMC11284034; doi:10.3389/fimmu.2024.1418290)
Supplement: Supplementary Table 1 — Expression of Nlrp6 and NLRP6 in various cell lines. qRT-PCR analysis of Nlrp6 and NLRP6 expression in indicated mouse and human cell1lines. The table showed the raw CT value. All the results are representative of at least 3 independent experiments. [file DataSheet_1.pdf]

Supplementary Table 1

Target: *Nlrp6*			
Sample	**CT**		
3T3	ND	34.97	ND
Raw264.7	ND	34.84	36.92
J774	34.76	36.47	34.71
myc-macrophage	35.82	ND	ND
BMDM	ND	ND	ND
Progenitors cell line	ND	37.88	38.08
Hepa 1-6	32.18	32.07	32.75
CMT93	ND	ND	ND

Target: *NLRP6*			
Sample	**CT**		
293T	39.11	41.03	40.93
A549	35.30	35.31	34.13
HeLa	41.35	41.08	ND
CaCO2	33.53	33.41	33.13
T84	32.10	31.78	32.00
HL60	31.48	31.48	31.48
THP1	29.08	29.00	29.11
U937	30.66	30.77	30.80

ND: not detectable
